# Spatiotemporal characterization of extracellular matrix maturation in human artificial stromal-epithelial tissue substitutes

**DOI:** 10.1186/s12915-024-02065-y

**Published:** 2024-11-18

**Authors:** Paula Ávila-Fernández, Miguel Etayo-Escanilla, David Sánchez-Porras, Ricardo Fernández-Valadés, Fernando Campos, Ingrid Garzón, Víctor Carriel, Miguel Alaminos, Óscar Darío García-García, Jesús Chato-Astrain

**Affiliations:** 1 Tissue Engineering Group, Department of Histology, University of Granada, Avenida Doctor Jesús Candel Fábregas, 11, E18016, Granada, Spain; 2 Instituto de Investigación Biosanitaria ibs.GRANADA, Granada, Spain; 3 Doctoral Program in Biomedicine, University of Granada, Granada, Spain; 4 Division of Pediatric Surgery, University Hospital Virgen de Las Nieves, Granada, Spain

**Keywords:** Extracellular matrix, Artificial tissue substitutes, Tissue engineering, Epithelial-mesenchymal interaction, Matrix maturation, Basement membrane

## Abstract

**Background:**

Tissue engineering techniques offer new strategies to understand complex processes in a controlled and reproducible system. In this study, we generated bilayered human tissue substitutes consisting of a cellular connective tissue with a suprajacent epithelium (full-thickness stromal-epithelial substitutes or SESS) and human tissue substitutes with an epithelial layer generated on top of an acellular biomaterial (epithelial substitutes or ESS). Both types of artificial tissues were studied at sequential time periods to analyze the maturation process of the extracellular matrix.

**Results:**

Regarding epithelial layer, ESS cells showed active proliferation, positive expression of cytokeratin 5, and low expression of differentiation markers, whereas SESS epithelium showed higher differentiation levels, with a progressive positive expression of cytokeratin 10 and claudin. Stromal cells in SESS tended to accumulate and actively synthetize extracellular matrix components such as collagens and proteoglycans in the stromal area in direct contact with the epithelium (zone 1), whereas these components were very scarce in ESS. Regarding the basement membrane, ESS showed a partially differentiated structure containing fibronectin-1 and perlecan. However, SESS showed higher basement membrane differentiation, with positive expression of fibronectin 1, perlecan, nidogen 1, chondroitin-6-sulfate proteoglycans, agrin, and collagens types IV and VII, although this structure was negative for lumican. Finally, both ESS and SESS proved to be useful tools for studying metabolic pathway regulation, revealing differential activation and upregulation of the transforming growth factor-β pathway in ESS and SESS.

**Conclusions:**

These results confirm the relevance of epithelial-stromal interaction for extracellular matrix development and differentiation, especially regarding basement membrane components, and suggest the usefulness of bilayered artificial tissue substitutes to reproduce ex vivo the extracellular matrix maturation and development process of human tissues.

**Graphical Abstract:**

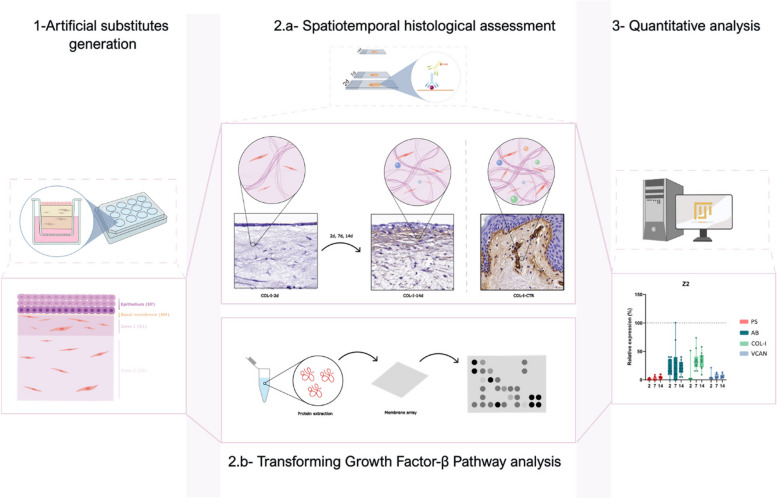

**Supplementary Information:**

The online version contains supplementary material available at 10.1186/s12915-024-02065-y.

## Highlights

1- Artificial bilayered substitutes developed by tissue engineering allow the spatiotemporal study of extracellular matrix maturation and development.

2- Epithelial tissue substitutes generated on the surface of acellular biomaterials display limited differentiation levels, with limited expression of relevant extracellular matrix components.

3- Full-thickness stromal-epithelial substitutes with a cellular stroma showed significantly higher levels of maturation and differentiation, with higher expression of epithelial (cytokeratin 10, claudin) and stromal markers (collagens, proteoglycans).

4- For the formation and development of the basement membrane, an interaction process between the epithelial and the stromal cells is necessary for the synthesis of most fibrillar and non-fibrillar components of this structure.

5- Our results confirm the intrinsic capability of epithelial cells to synthesize extracellular matrix components and the positive inductive effect of paracrine factors released by stromal cells.

**Supplementary Information:**

The online version contains supplementary material available at 10.1186/s12915-024-02065-y.

## Background

The extracellular matrix (ECM) consists of a macromolecular network of proteins, fibers, and signaling molecules and serves as the architectural scaffold and dynamic microenvironment for cells, modulating cellular behavior and providing biomechanical features within tissues [1, 2]. In the human connective tissues, collagen and non-collagen molecules, such as proteoglycans, glycoproteins, and glycosaminoglycans, constitute the vast majority of the ECM components. Collagen molecules within the ECM play essential roles in determining its mechanical properties, structural organization, and overall functional capabilities. Meanwhile, non-fibrillar components such as proteoglycans, glycosaminoglycans, and glycoproteins are pivotal for facilitating interactions between cells and the ECM. They regulate cellular behavior by transmitting biochemical signals and contribute to the physical properties of the ECM through their interactions with fibrillar components [3]. These ECM molecules and their interactions with cellular components orchestrate and guide tissue repair, maintenance, and regeneration processes [1, 4–6]. Histologically, bilayered tissues and organs such as the human skin, cornea, oral mucosa, and urothelium consist of two well differentiated layers: a superficial epithelial tissue and a profound connective tissue known as stroma containing a dense ECM in which fibroblasts and other cells reside. The epithelial and the stromal tissue layers are connected by the basement membrane (BM), a specialized structure anchoring the epithelial layer while promoting cellular cross-talk between both layers [7], and it has been demonstrated that the stromal layer plays an important role in supporting and inducing epithelial differentiation and function [8].

The dynamic nature of the human ECM allows controlled remodeling and adaptation to physiological demands. In this sense, advances in tissue engineering have provided new strategies for exploring these complex processes through controlled and reproducible experimental models that could contribute to reveal ECM maturation and differentiation and the interplay between cell behavior and matrix deposition. Understanding ECM maturation is essential for tissue development, wound healing, regenerative medicine, and artificial tissues emerge as promising tools to investigate these processes and facilitate their understanding [6].

In previous works, we described a dermo-epidermal model of human bioartificial skin that demonstrated usefulness for the treatment of severely burned patients [9, 10]. This model, called UGRSKIN, was generated using a fibrin-agarose scaffold which uses the high biocompatibility of human plasma while enhancing biomechanical properties through the incorporation of a small percentage of agarose, a marine-derived algae product. In this hydrogel, fibroblasts were immersed within, and keratinocytes were allowed to generate an epithelium on top [11]. This skin substitute demonstrated in previous studies its potential to biomimetically replicate the histoarchitecture and differentiation patterns of normal human skin both ex vivo and in animal models [12, 13]. In the same line, and based on the cited fibrin-agarose hydrogel, bilayered tissue models of the human cornea, oral mucosa, and urinary mucosa previously generated by our research group also demonstrated their potential to reproduce the differentiation patterns of native tissues, providing a good model to carry out ex vivo time-course studies of cell and tissue differentiation at the epithelial and stromal layers [14–16].

The interaction between epithelial tissues and the underlying stroma is complex and depends on the structure and function of the BM [7]. The complexity of the BM structure enables permeation and diffusion of selective molecules in native tissues and constitutes a physical support and a key element for cellular differentiation, proliferation, and migration [17]. BM is unique in terms of structure and composition and consists of a complex mixture of fibrillar components, such as collagens types IV (COL-IV) and VII (COL-VII), and different non-fibrillar molecules, including nidogen 1 (NID1), chondroitin-6-sulfate proteoglycans (CH6S), agrin (AGRN), and perlecan (HSPG2), lumican (LUM), and fibronectin 1 (FN1), among others [16–18]. Despite its relevance, there is nowadays a gap of knowledge in BM development, especially in human tissues generated by tissue-engineering [6].

In this study, we carried out a comprehensive spatiotemporal histological analysis of the ECM maturation process that takes place in a human bilayered tissue substitute. By characterizing the progressive synthesis of fibrillar and non-fibrillar ECM components, we studied the sequence of ECM molecule expression and the role of the epithelial and stromal cells in this process. Here, we generated bilayered human tissue substitutes consisting of a cellular connective tissue with a suprajacent epithelium (full-thickness stromal-epithelial substitutes or SESS) and human tissue substitutes with an epithelial layer generated on top of an acellular biomaterial (epithelial substitutes or ESS). Both types of artificial tissues were studied at sequential time periods (2, 7, and 14 days) to analyze the maturation process of the ECM using histochemical and immunohistochemical techniques. Our findings may contribute not only to understanding ECM dynamics in artificial bilayered substitutes but also in the ECM maturation process.

## Results

### Histological analysis of the human bilayered tissue substitutes using hematoxylin and eosin staining

**Fig. 1:**
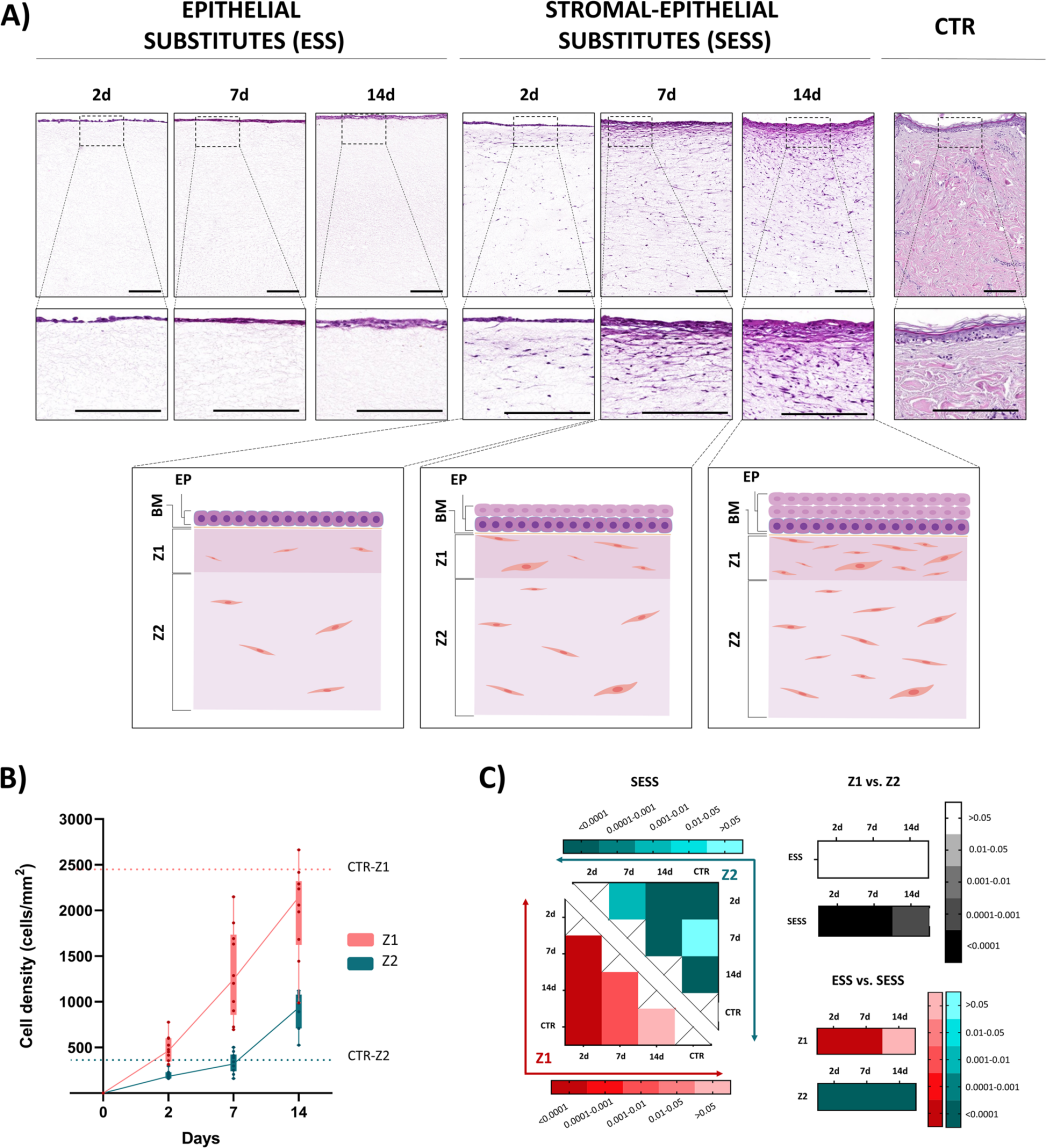
Histological evaluation of the epithelial substitutes (ESS) and full-thickness stromal-epithelial substitutes (SESS). **A** Histological evaluation of the human artificial tissues stained with hematoxylin–eosin (HE) at different magnifications. Bottom images correspond to schematic illustrations of each follow-up time of SESS. EP: epithelium, BM: basement membrane, Z1: zone 1 of the stromal layer, Z2: zone 2 of the stromal layer. Scale bar = 200 μm. **B** Quantitative analysis of cell density at Z1 and Z2 at each follow-up time in SESS shown as number of cells per mm^2^ of stroma. **C** Heatmaps showing the statistical analysis of cell density at Z1 (reds) and Z2 (blues) for each follow-up time in SESS. Additionally, the heatmaps display *p*-values of the statistical comparison between Z1 and Z2 in black for each model (ESS and SESS) and time point as well as the comparison of ESS versus SESS for each zone (Z1 in reds and Z2 in blues) and time point. Values with *p* < 0.001 were considered statistically significant. The darker the color, the lower the *p*-value

Histological evaluation of the human artificial tissues generated in the present work stained with hematoxylin–eosin (HE) revealed the presence of an epithelium on top of the fibrin-agarose biomaterial in both ESS and SESS, with several differences related to the type of sample and the culture time (Fig. 1). As shown in Fig. 1A, the epithelial layer of ESS tended to mature and stratify with the culture time, showing a transition from a single-cell simple epithelium at day 2 of development to a stratified epithelium with 3–4 cell strata after 14 days of follow-up. As expected in the ESS, the biomaterial underlying the epithelium contained no cells and did not vary among the study time periods. In turn, SESS exhibited notable differences compared to ESS, both at the epithelial and the stromal layers. First, we found that the number of cell strata at the epithelial layer ranged from approximately 2 cell strata at day 2 of development to 4–5 strata at day 14, with cells tending to show a flattened morphology. Then, we found that the stromal layer of SESS displayed two distinct zones according to the distribution pattern of the stromal cells: zone 1 (Z1), immediately below the epithelium, and zone 2 (Z2), below Z1, corresponding to the majority of the artificial stroma. When the cell density was quantified at each zone, we found that Z1 contained higher cell density than Z2 at all time points (*p* < 0.001 at 2, 7, and 14 days) (Fig. 1B, C and Additional File 1: Table S1), and both, Z1 and Z2, exhibited a significant increase in cell density over time (*p* > 0.0001 and *r* = 0.7316 in Z1 and *p* > 0.0001 and *r* = 0.7635 in Z2 for the Kendall *tau* correlation test, Additional File 2: Table S2). The number of cells found at this stromal layer was comparable to control of human skin (*p* > 0.001) after 7 days in Z1 and only in 7 days at Z2. Nevertheless, while Z1 cell density remained comparable to control skin for 14 days, cell density significantly increased in Z2 (*p* < 0.001) (Fig. 1C and Additional File 2: Table S2).

### Phenotypic characterization of epithelial and stromal cells in the human bilayered tissue substitutes

**Fig. 2:**
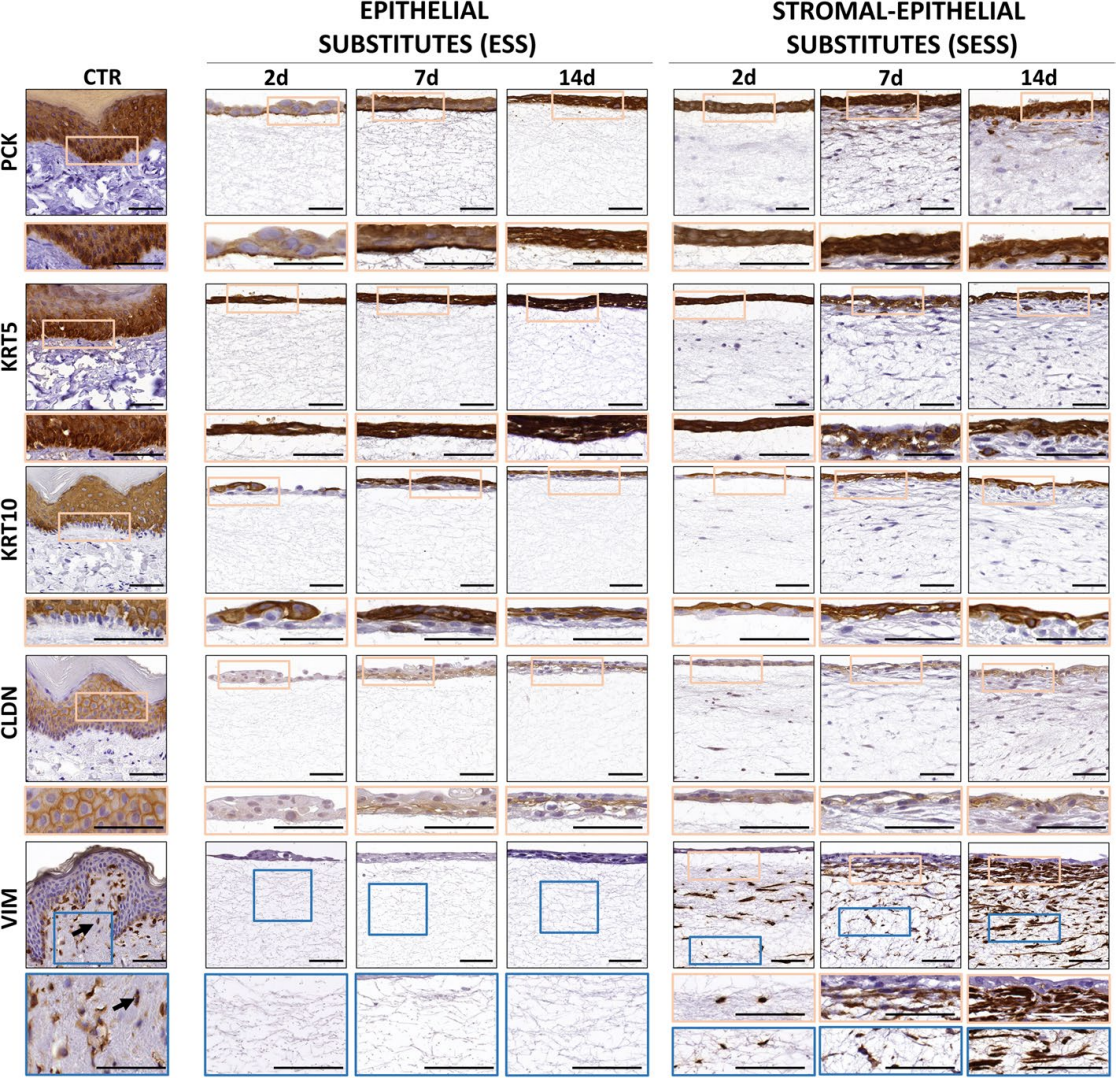
Immunohistochemical analysis of the cell profile in epithelial substitutes (ESS) and full-thickness stromal-epithelial substitutes (SESS). Immunohistochemical signal for pancytokeratin (PCK), cytokeratin 5 (KRT5), cytokeratin 10 (KRT10), claudin (CLDN) and vimentin (VIM) at various time points (2, 7, and 14 days) in each tissue sample. Scale bar = 50 μm

**Fig. 3:**
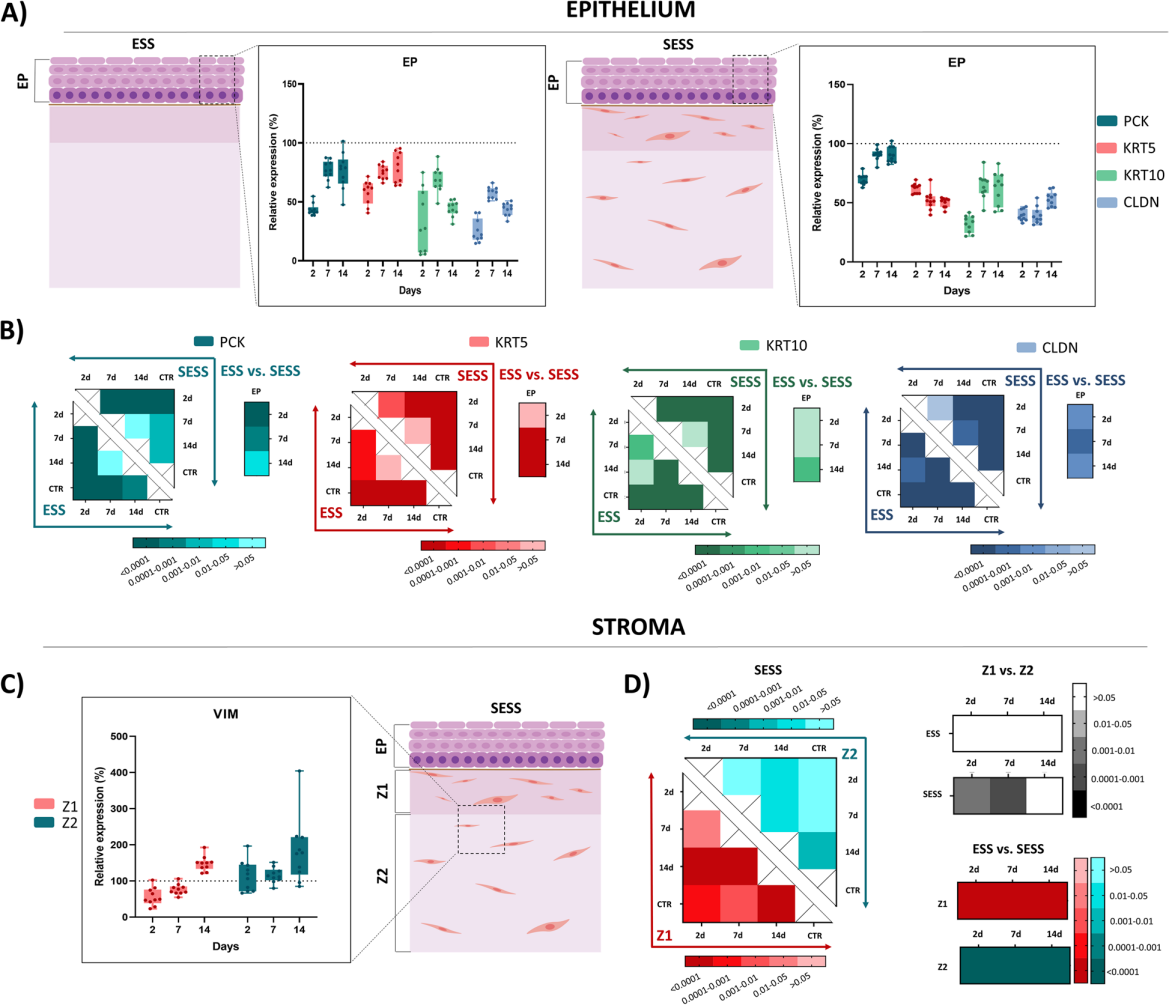
Immunohistochemical quantification and statistical analysis of the cell profile in epithelial substitutes (ESS) and full-thickness stromal-epithelial substitutes (SESS). **A** Immunohistochemical quantification for pancytokeratin (PCK), cytokeratin 5 (KRT5), cytokeratin 10 (KRT10), and claudin (CLDN) at various time points (2, 7, and 14 days) in each tissue sample. Values corresponding to the control tissues are shown as dotted lines. **B** Heatmaps showing the statistical analysis of pancytokeratin (PCK), cytokeratin 5 (KRT5), cytokeratin 10 (KRT10), and claudin (CLDN) expression in ESS and SESS models at each follow-up time, along with heatmaps displaying the statistical differences between ESS and SESS for each time point. The darker the color, the lower the *p*-value. **C** Immunohistochemical quantification for vimentin (VIM) at various time points (2, 7, and 14 days) in zone 1 (Z1) and zone 2 (Z2) of SESS. Values corresponding to the control tissues are shown as dotted lines. **D** Heat maps showing the statistical analysis of vimentin (VIM) at Z1 (reds) and Z2 (blues) for each follow-up time in SESS. Additionally, the heatmaps display *p*-values of the statistical comparison between Z1 and Z2 in black for each model (ESS and SESS) and time point as well as the comparison of ESS versus SESS for each zone (Z1 in reds and Z2 in blues) and time point. Values with *p* < 0.001 were considered statistically significant. The darker the color, the lower the *p*-value

To characterize the epithelial layer of ESS and SESS, we first analyzed the expression of several human cytokeratins. As shown in Fig. 2, results show a positive immunohistochemical reaction for pancytokeratin (PCK) and several cytokeratins in the epithelium of all samples, although differences were found among sample types. The staining signal significantly increased from day 2 to day 7 of follow-up for PCK and cytokeratin 5 (KRT5) in ESS, with a significant correlation with time (*p* = 0.0002, *r* = 0.5383 for PCK and *p* = 0.0004, *r* = 0.5216 for KRT5), and for PCK and cytokeratin 10 (KRT10) in SESS, with a significant correlation with time (*p* = 0.0006, *r* = 0.5057 for PCK and *p* = 0.0003, *r* = 0.5333 for KRT10) (Fig. 3A and Additional File 2: Table S2). Interestingly, SESS displayed a negative KRT5 staining signal from 2 days onwards with a significant correlation (*p* = 0.0003. *r* = − 0.5393) (Fig. 3A and Additional File 2: Table S2). The signal corresponding to PCK, KRT5 and KRT10 was significantly lower in the bioartificial tissue substitutes than in the control group (CTR) at all times, except for PCK at days 7 and 14, which reached the levels of the CTR (Fig. 3B and Additional File 2: Table S2). In the second place, epithelial cells were characterized for the intercellular junction protein claudin (CLDN). Although the immunohistochemical staining signal was not able to reach the levels of the CTR in any of the study groups (*p* < 0.001 for all comparisons with CTR), we found that CLDN expression significantly increased from day 2 to day 14 of follow-up, although correlation with time did not reach statistical significance (Fig. 3B and Additional File 2: Table S2).

In addition, characterization of the stromal cell population was carried out by immunohistochemistry for the stromal marker vimentin (VIM). In this regard, our findings suggest that the number of VIM-positive cells did not differ between SESS and CTR tissues at Z2, whereas significant differences were detected between SESS and CTR (Fig. 3C, D, data shown in Additional File 2: Table S2) in Z1 at 2 days, which was lower than CTR (*p* = 0.0005), and day 14, which was higher than CTR (*p* < 0.0001), but not at day 7, which was comparable to CTR. Correlation with the time in culture was significant only for Z1 (*p* < 0.0001, *r* = 0.7150, see in Additional File 2: Table S2).

### Analysis of cell proliferation and apoptosis of epithelial and stromal cells in the human bilayered tissue substitutes

**Fig. 4:**
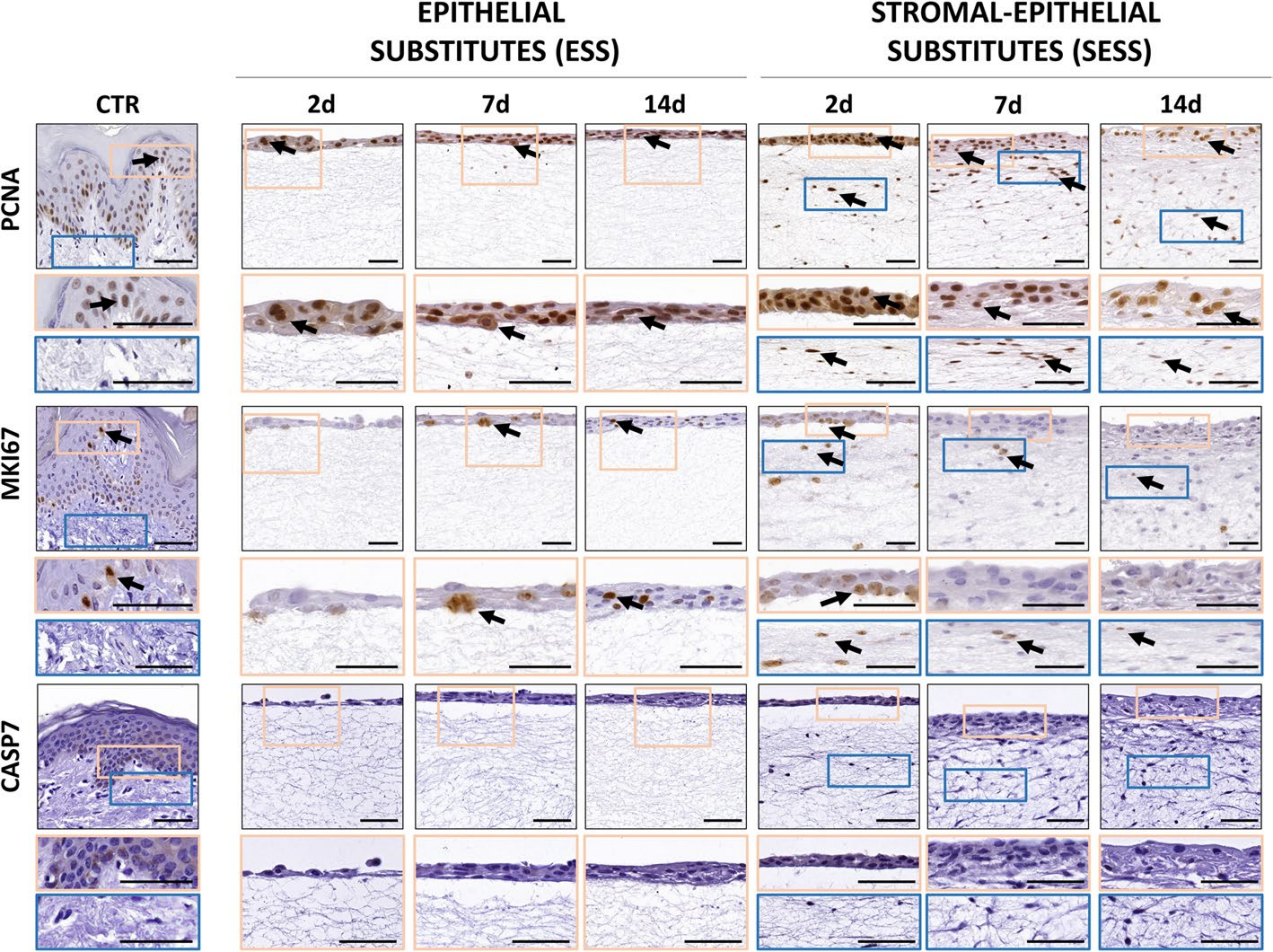
Assessment of the proliferative and apoptotic status in epithelial substitutes (ESS) and full-thickness stromal-epithelial substitutes (SESS). Immunohistochemical signal for PCNA, MKI67, and caspase 7 (CASP7) at various time points (2, 7, and 14 days) in each tissue sample. Black arrows highlight illustrative immunohistochemical positive reactions. Scale bar = 50 μm

**Fig. 5:**
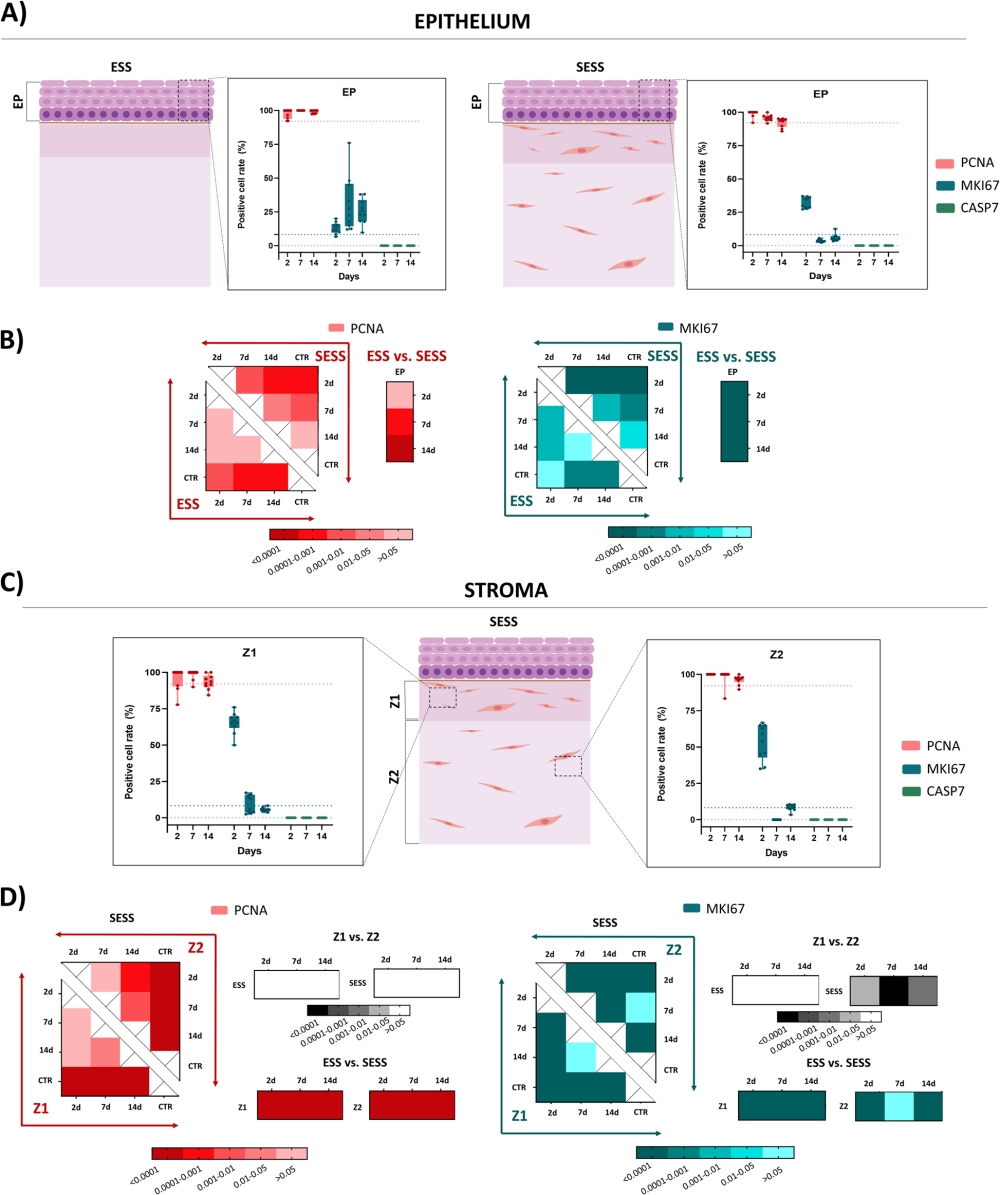
Quantification and statistical analysis of the proliferative and apoptotic status in epithelial substitutes (ESS) and full-thickness stromal-epithelial substitutes (SESS). **A** Quantitative analysis results of the epithelium proliferative rates, expressed as the percentage of positively stained cells. Values corresponding to the control tissues are shown as dotted lines. **B** Heatmaps showing the statistical analysis of PCNA and MKI67 immunohistochemical epithelium quantification in ESS and SESS models at each follow-up time, along with heatmaps displaying the statistical differences between ESS and SESS for each time point. **C** Quantitative analysis results of the stroma proliferative rates, expressed as the percentage of positively stained cells. **D** Heat maps showing the statistical analysis of PCNA (reds) and MKI67(blues) at zone 1 (Z1) and zone 2 (Z2) for each follow-up time in SESS. Additionally, the heatmaps display *p*-values of the statistical comparison between Z1 and Z2 in black for each model (ESS and SESS) and time point as well as the comparison of ESS versus SESS for each zone (Z1 and Z2) and time point. Values with *p* < 0.001 were considered statistically significant. The darker the color, the lower the *p*-value

In order to evaluate the proliferation status of the different cell types found in the ESS and SESS tissues, we carried out immunohistochemical analyses of PCNA and MKI67 expression (Fig. 4). At the epithelial level, our results showed a positive reaction for PCNA in most epithelial cells of both the ESS and SESS, along with CTR tissues, whereas MKI67 signal was mostly localized at the basal layer of the epithelium of both tissue types, and CTR. For PCNA, the number of positive cells in ESS was comparable to CTR tissues at 2 days of culture, and the correlation of PCNA positivity with the culture time was not statistically significant (Fig. 5A, B and Additional File 2: Table S2). In contrast, a significant negative correlation with time was found in SESS (*p* < 0.0001, *r* = − 0.6232), suggesting that this marker tended to decrease with the culture time (Fig. 5B, and Additional File 2: Table S2).

Differences between ESS and SESS were statistically significant at days 7 and 14 of follow-up (Fig. 5B). For MKI67, the number of positive cells was lower than for PCNA, and the correlation with the time in culture was not statistically significant. As can be seen in Fig. 5B, differences with CTR were non-significant after 14 days in SESS (see Additional File 1: Table S1). Differences between ESS and SESS were statistically significant at all comparison times.

When the stromal layer was analyzed, we found that both the Z1 and the Z2 zones of SESS contained significantly higher percentages of PCNA-positive cells than CTR tissues at all the study times, with non-significant differences between Z1 and Z2 (Fig. 5D, Additional File 2: Table S2). A significant negative correlation with the culture time was found only at Z2 (*p* = 0.0001, *r* = − 0.6320) (Additional File 2: Table S2). For MKI67, results showed a significantly higher percentage of positive cells in Z1 and Z2 as compared to CTR at all times, except for Z2 at day 7 of development, and the correlation with time was only significant for Z1 (*p* < 0.0001, *r* = − 0.6070), but not for Z2. This percentage was significantly higher in Z1 than Z2 after 7 days of follow-up (*p* < 0.0001).

In addition, we evaluated the percentage of cells undergoing apoptotic cell death by using caspase 7 (CASP7) immunohistochemistry. As shown in Figs. 4 and 5A, results were negative in controls and in the epithelial and stromal cells of ESS and SESS at all the follow-up times.

### Histochemical and immunohistochemical assessment of ECM components in the stromal layer of human bilayered tissue substitutes

**Fig. 6:**
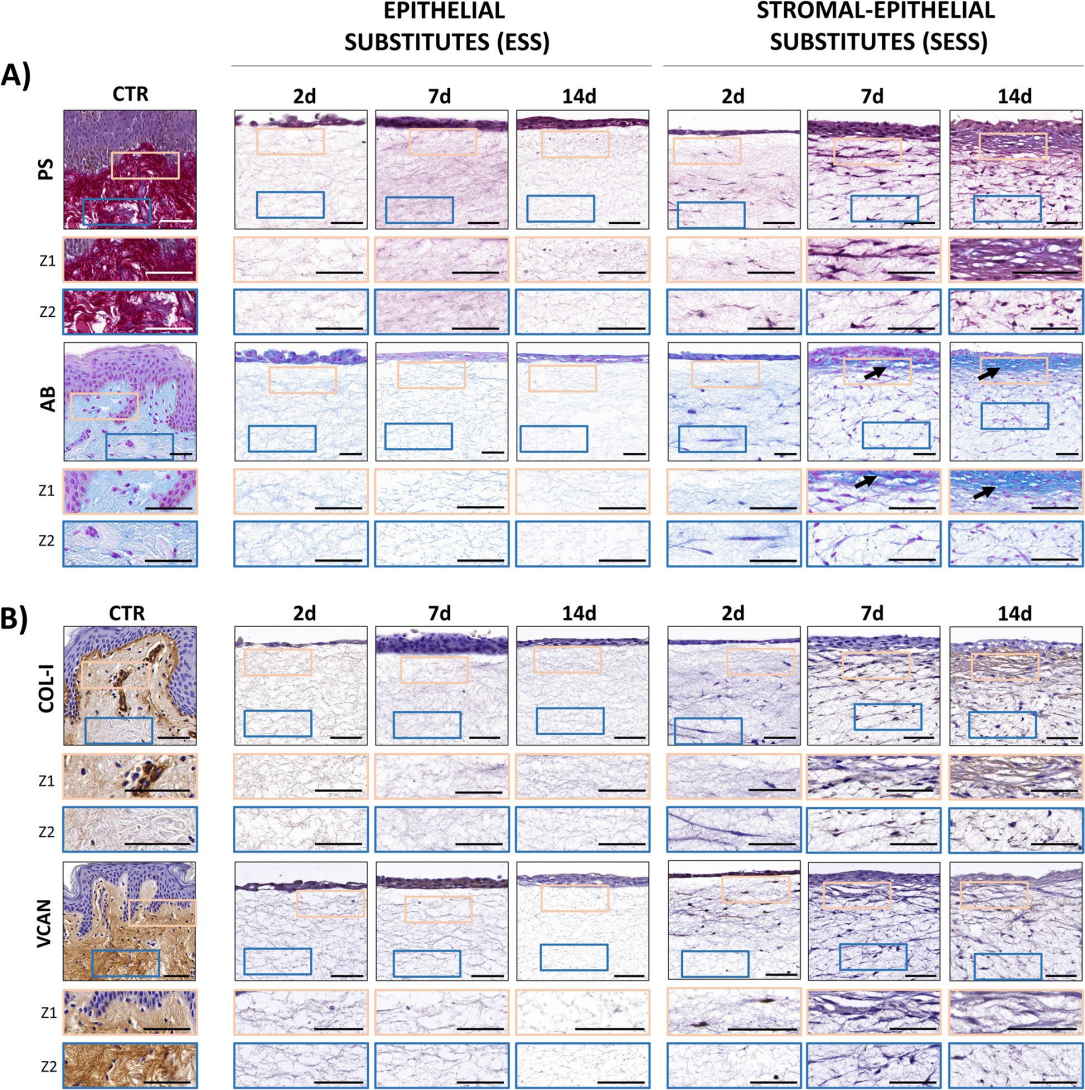
Assessment of extracellular matrix (ECM) components in epithelial substitutes (ESS) and full-thickness stromal-epithelial substitutes (SESS). **A** Histochemical signal for picrosirius red (PS) and alcian blue (AB) staining. **B** Immunohistochemical staining for collagen type I (COL-I) and versican (VCAN). Illustrative areas of positive signal are highlighted with black arrows. In **A** and **B**, images are shown at various time points (2, 7, and 14 days) in each tissue sample, and high-magnification areas corresponding to zone 1 (Z1) and zone 2 (Z2) are shown in inserts below each image (orange and blue, respectively). Scale bar = 50 μm

**Fig. 7:**
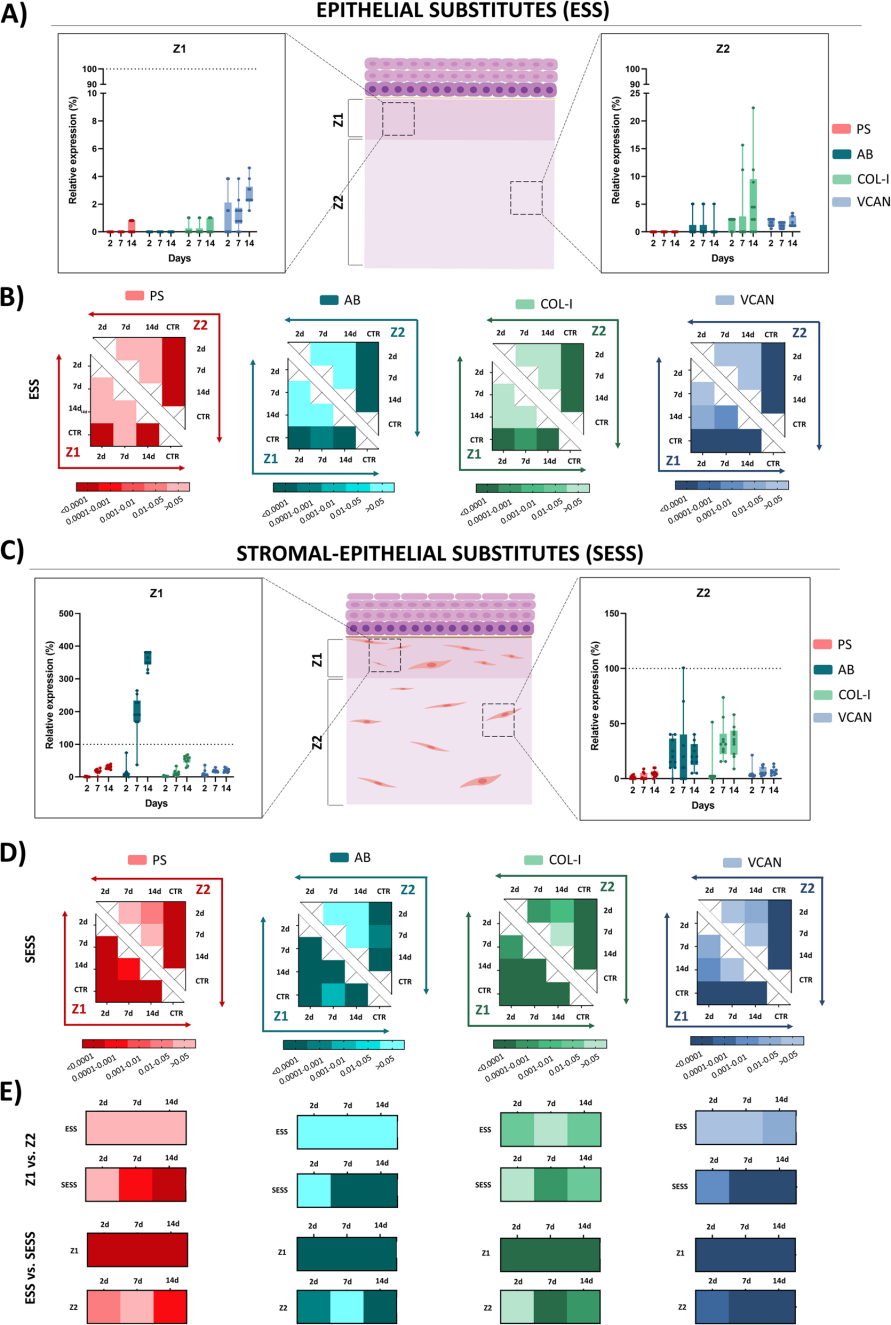
Quantification and statistical analysis of the extracellular matrix (ECM) components expression in epithelial substitutes (ESS) and full-thickness stromal-epithelial substitutes (SESS). **A** Quantitative analysis results of the histochemical and immunohistochemical reactions for picrosirius red (PS), alcian blue (AB), collagen type I (COL-I) and versican (VCAN) in ESS model. **B** Heatmaps showing the statistical analysis of PS, AB, COL-1 and VCAN expression intensity at zone 1 (Z1) and zone 2 (Z2) for each follow-up time in ESS. **C** Quantitative analysis results of the histochemical and immunohistochemical reactions for PS, AB, COL-I, and VCAN in the SESS model. **D** Heatmaps showing the statistical analysis of PS, AB, COL-1, and VCAN expression intensity at Z1 and Z2 for each follow-up time in SESS. **E** Heatmaps showing the statistical analysis of PS, AB, COL-1, and VCAN expression intensity between Z1 and Z2 for each model (ESS and SESS) and time point as well as the comparison of ESS versus SESS for each zone (Z1 and Z2) and time point. Values with *p* < 0.001 were considered statistically significant. The darker the color, the lower the *p*-value

In order to shed light on the sequential process of ECM maturation that takes place in the stroma of the bioartificial bilayered tissues, we first analyzed the presence of several collagen fibers in each experimental group (Fig. 6). On the one hand, picrosirius red (PS) histochemical staining quantification revealed that the ESS stroma was mostly devoid of collagen fibers identified by this method (Fig. 7A, B). However, SESS showed a positive PS signal, especially at Z1, which was significantly higher than Z2 at days 7 (*p* = 0.0001) and 14 (*p* < 0.0001), although the PS signal was significantly lower than CTR in all bioartificial samples (Z1 and Z2 at all follow-up times) (Fig. 7C and 7D, Additional File 2: Table S2). A positive significant correlation with time was found in Z1 (*p* < 0.0001, *r* = 0.7984), but not in Z2. Then, we analyzed the presence of collagen type I (COL-I) by immunohistochemistry (Fig. 6). Results of this analysis showed that COL-I was not present in ESS, but it was detectable in SESS. A significant correlation with the culture time was found (*p* < 0.0001, *r* = 0.8158 for Z1 and *p* = 0.0007, *r* = 0.5069 for Z2), with a progressive increase at days 7 and 14 of development, although levels were always significantly lower than CTR tissues (*p* < 0.0001 for Z1 and Z2 at all times).

In the second place, we assessed the presence of non-fibrillar components of the ECM at the stromal level using alcian blue (AB) histochemistry and versican (VCAN) immunohistochemistry. Results show that the ESS stroma was mostly devoid of the presence of proteoglycans identified by both methods, although a positive signal was detected in SESS (Fig. 7A, C, respectively, data shown in Additional File 2: Table S2) specially using AB whose signal intensity was comparable to CTR at 7 days (*p* = 0.002) and even higher at 14 days (*p* < 0.0001) in Z1. Moreover, we found that these non-fibrillar ECM components were significantly more abundant in Z1 than in Z2 in SESS samples corresponding to 7 and 14 days of development (*p* < 0.0001) for both staining methods (AB and VCAN). Correlation with the culture time was significant only for Z1 stained with AB (*p* < 0.0001, *r* = 0.8476).

### Histochemical and immunohistochemical assessment of basement membrane components in human bilayered tissue substitutes

**Fig. 8:**
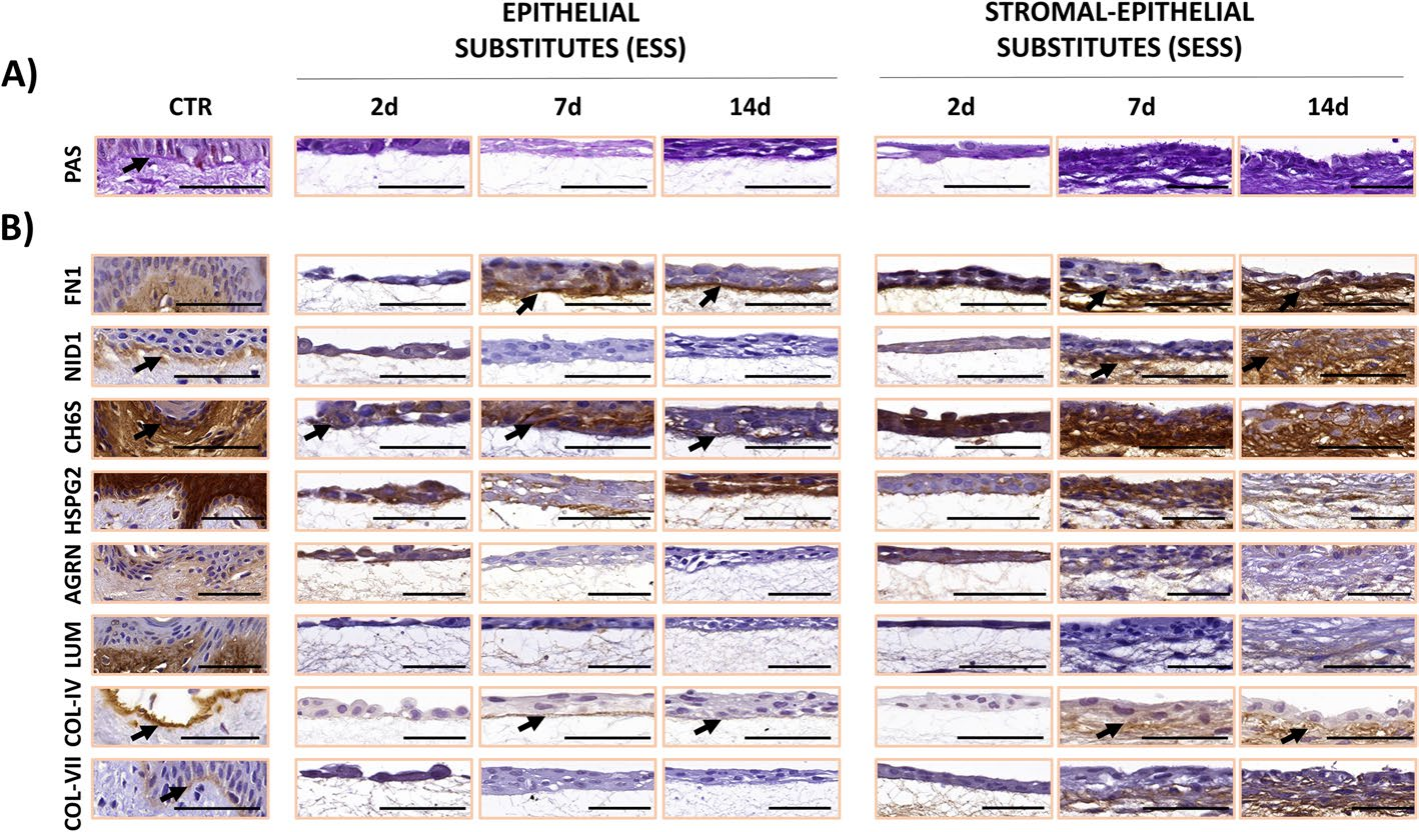
Histological analysis of the basement membrane (BM) in epithelial substitutes (ESS) and full-thickness stromal-epithelial substitutes (SESS). **A** Histochemical identification of the BM using periodic acid–Schiff (PAS) staining. **B** Immunohistochemical identification of key BM components, including fibronectin 1 (FN1), nidogen 1 (NID1), chondroitin-6-sulfate proteoglycans (CH6S), perlecan (HSGP2), agrin (AGRN), lumican (LUM), and collagens types IV (COL-IV) and VII (COL-VII). In **A** and **B**, results are shown at various time points (2, 7, and 14 days) in each tissue sample. Black arrows highlight illustrative histochemical positive reactions. Scale bar = 50 μm

**Fig. 9:**
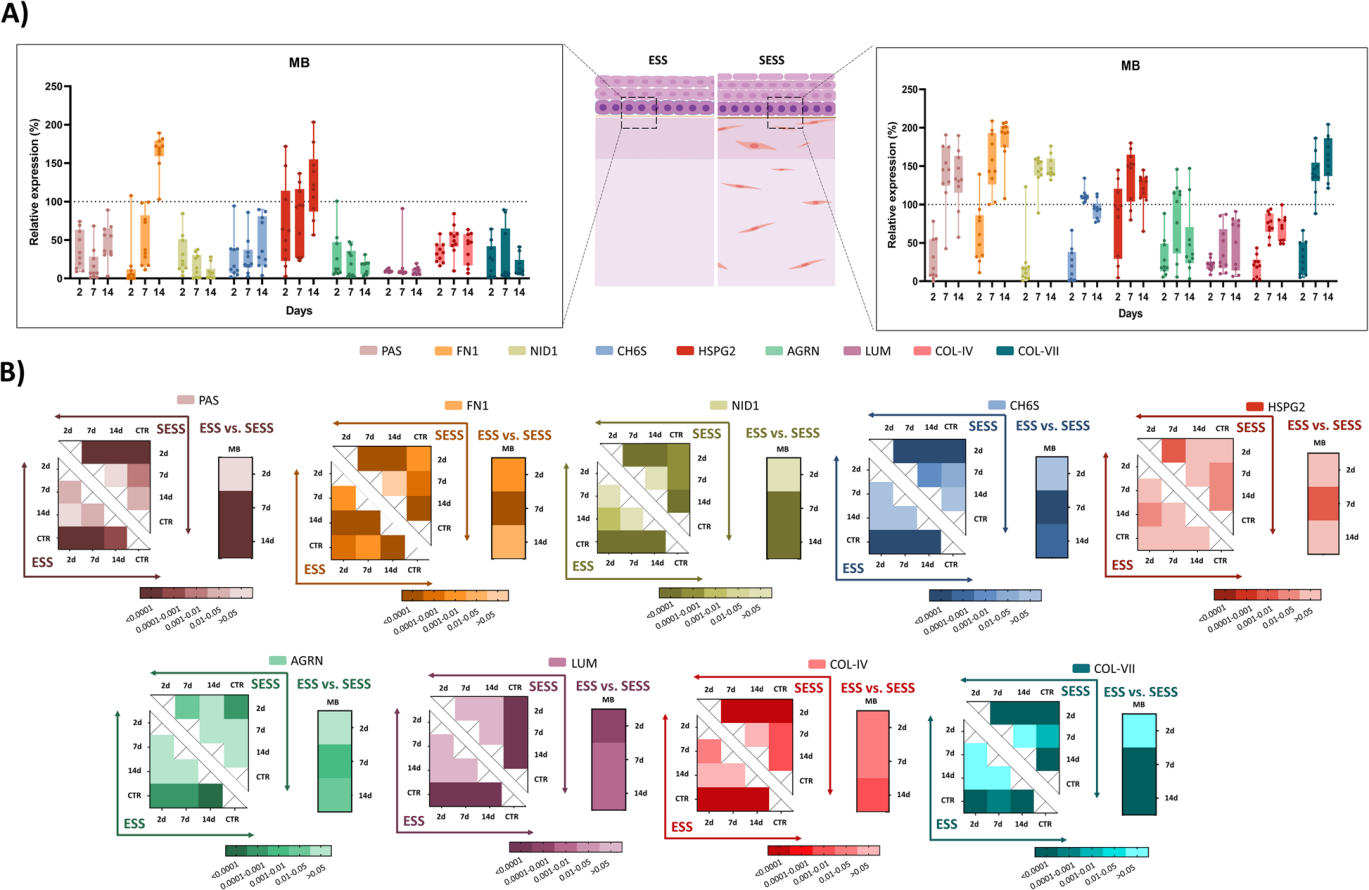
Quantification and statistical analysis of the basement membrane (BM) components expression in epithelial substitutes (ESS) and full-thickness stromal-epithelial substitutes (SESS). **A** Quantitative analysis results of periodic acid-Schiff (PAS), fibronectin 1 (FN1), nidogen 1 (NID1), chondroitin-6-sulfate proteoglycans (CH6S), perlecan (HSGP2), agrin (AGRN), lumican (LUM), and collagens types IV (COL-IV) and VII (COL-VII) reactions, normalized to control group (CTR) tissues (whose signal was considered as 100%). Values corresponding to the CTR tissues are shown as dotted lines. **B** Heatmaps showing the statistical analysis of PAS, FN1, NID1, CH6S, HSPG2, AGRN, LUM, COL-IV, and COL-VII quantification in ESS and SESS models at each follow-up time, along with heatmaps displaying the statistical differences between ESS and SESS for each time point. Values were considered statistically significant below 0.001

With the objective of determining the presence of the main components of the human BM in the bilayered tissue substitutes, we first carried out a histochemical analysis using the periodic acid-Schiff (PAS) staining method to identify ECM glycoproteins (Fig. 8). As shown in Fig. 9A and B and in Additional File 2: Table S2, results show that the PAS staining signal was significantly lower than CTR in all ESS tissues and in SESS kept in culture for 2 days (*p* < 0.001) but reached the levels of the CTR tissues in SESS corresponding to 7 and 14 days of follow-up. Correlation with the culture time was statistically significant for SESS (*p* = 0.0005, *r* = 0.6074), but not for ESS.

Then, we analyzed several key non-fibrillar components of the BM using immunohistochemistry. For FN1, our results suggest that the signal intensity for this glycoprotein tended to increase with time, with a significant correlation with time in both the ESS (*p* < 0.0001, *r* = 0.7711) and SESS (*p* < 0.0001, *r* = 0.6651) (Additional File 2: Table S2). In fact, the signal was significantly lower in ESS at day 2 than in CTR, was comparable to CTR at day 7, and was significantly higher than CTR at day 14 of development. For SESS, the signal did not differ from the CTR at day 2 and became significantly higher than CTR at days 7 and 14 (Fig. 9B, Additional File 2: Table S2). For NID1, we found a significant correlation of the staining signal with the culture time only for SESS (*p* = 0.0002, *r* = 0.5389), but not for ESS, with this signal being significantly lower than CTR tissues in all ESS samples and in SESS corresponding to 2 days, whereas the signal was significantly higher than CTR in SESS kept in culture for 7 and 14 days. When the immunohistochemical expression of CH6S and AGRN was analyzed, we found that all ESS tissues and SESS samples at 2 days were significantly lower than CTR, with SESS at days 7 and 14 being statistically similar to CTR, although correlation with the culture time did not reach statistical significance (Fig. 9B). Then, the analysis of HSPG2 showed that all ESS and SESS samples were comparable to CTR, whilst LUM expression was significantly lower than CTR in all ESS and SESS bioartificial tissues, with no correlation with the culture time for HSPG2 or LUM.

Finally, the BM was characterized by determining the presence of two relevant fibrillar components of this structure (Figs. 8 and 9A, B, and Additional File 2: Table S2): COL-IV and COL-VII. In both cases, our results suggest that the presence of these types of collagens was significantly lower than CTR tissues in ESS at the three time points analyzed, and in SESS corresponding to 2 days of development (*p* < 0.001 for all these comparisons), whereas SESS at 7 days was similar to CTR for COL-IV and COL-VII and significantly higher at 14 days for COL-VII (*p* < 0.0001). For both types of collagens, a significant correlation with time was found for SESS (*p* = 0.0004, *r* = 0.5200 for COL-IV and *p* < 0.0001, *r* = 0.6742 for COL-VII), but not for ESS.

### Transforming growth factor-β pathway analysis in human bilayered tissue substitutes

**Fig. 10:**
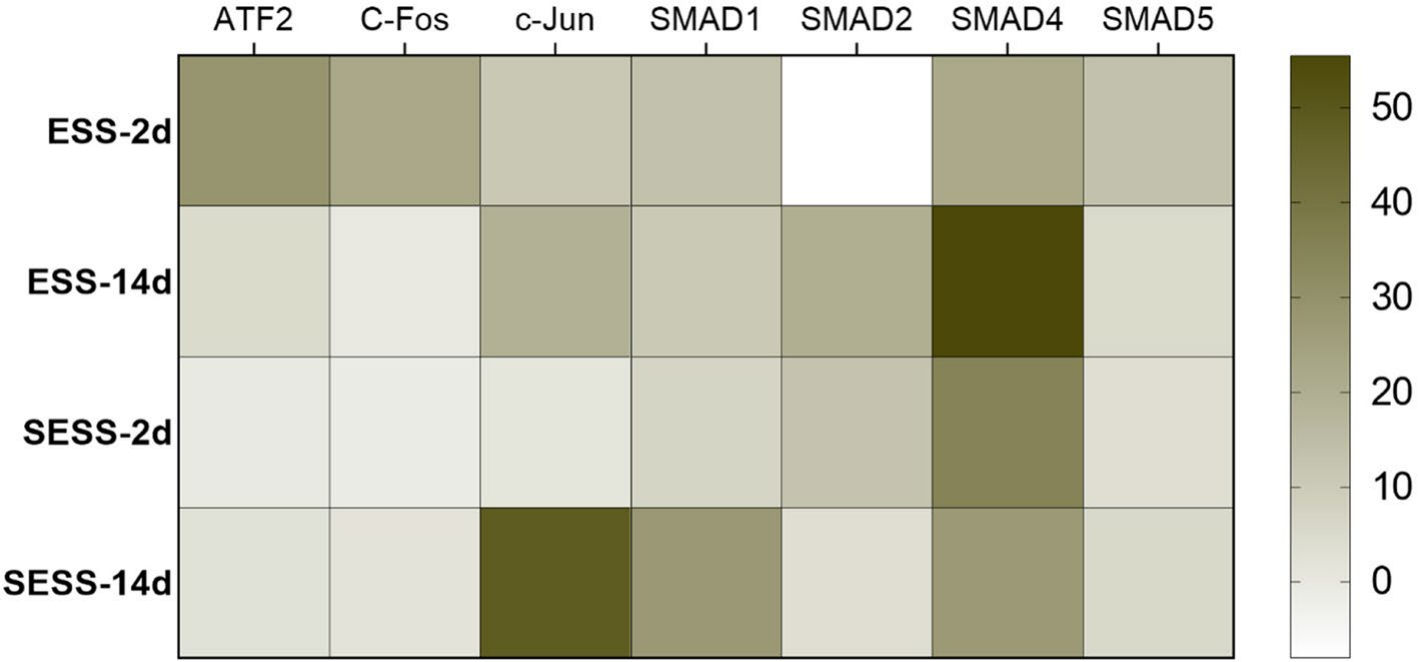
Protein array analysis of transforming growth factor-β (TGF-β) pathway activation in human bilayered tissue substitutes. Mean intensity expression of key TGF-β pathway proteins in epithelial substitutes (ESS) and full-thickness stromal-epithelial substitutes (SESS) at 7 and 14 days. Signal intensities were normalized against positive and negative controls integrated into the array

To study the mechanisms and investigate the signaling pathways that influence tissue maturation and ECM deposition, we performed a protein array analysis of transforming growth factor-β (TGF-β) pathway as shown in Fig. 10.

First, our results showed that our human bilayered tissue substitutes could be used to successfully investigate signaling pathways activation and thus the mechanistical insight the underly complex biological processes like tissue maturation. Specifically, our findings indicate that temporal progression remarkably influenced the activation of key metabolic pathways, including the TGF-β pathway, as evidenced by the increase in mean relative protein activation (Fig. 10).

Additionally, our results showed that the different cell types found in the ESS and SESS tissues distinctly modulates the activation of TFG-β metabolic pathway. In fact, in the ESS model, SMAD4 protein exhibited the highest levels of activation after 14 days. In contrast, the SESS model showed a different pattern of pathway activation. After 14 days, c-Jun protein was identified as the most activated protein. Additionally, there was notable activation of SMAD in SESS proteins as compared with 7 days, specifically SMAD1 and SMAD4.

## Discussion

For the generation of a successful tissue-engineered substitute, physical, chemical, and biological interactions must coordinate adequate cellular responses. In this sense, the ECM and, specially, the BM plays a vital role on cell fate, function, and physiological regulation in bilayered substitutes [7]. Understanding the process of ECM maturation is crucial for tissue development, wound healing and regenerative medicine [4, 6]. Despite its importance, ECM and BM generation and remodeling in artificial substitutes is poorly understood [6]. In this line, tissue-engineered substitutes offer a controlled and simplified model for studying the complex biological mechanisms underlying ECM maturation [18]. To the best of our knowledge, this is one of the first studies investigating the spatiotemporal dynamics of ECM maturation focused on the BM formation in human bilayered tissue substitutes.

First, this study demonstrated that bilayered artificial tissue substitutes can be used as a controlled and reproducible experimental system able to mimic normal human skin tissues, as previously reported [11, 12, 19]. On the one hand, we found that ESS exhibited a partial development of the epithelial layer, evolving from a single-cell layer after 2 days of development to a stratified epithelium after 14 days, with a correlation with the culture time only for some markers such as PCK and KRT5. These findings are in agreement with previous reports suggesting that epithelial substitutes devoid of a cellular stroma may partially develop an epithelial layer on top, although this epithelium is typically, poorly differentiated [20, 21]. On the other hand, our analysis of the SESS epithelium showed higher levels of epithelial maturation and differentiation, with more flattened epithelial cells from day 2 of development, suggesting that the inclusion of fibroblasts in the stromal layer may contribute to a more mature epithelial phenotype in different types of bilayered tissues [22]. This idea was confirmed by the expression results of cytokeratins, essential epithelial structural proteins, which exhibited a more mature phenotype profile in SESS than in ESS, potentially influenced by the presence of fibroblasts in the stroma. Interestingly, we found that KRT5 expression, which is typically associated to proliferative, basal epidermal strata in human epithelia [23], tended to decrease from 7 days to 14 days in SESS, whilst KRT10 expression, which is associated to suprabasal mature and terminally differentiated strata [24], tended to increase with culture time. In contrast, ESS showed the opposite behavior, implying that these substitutes could be partially undifferentiated, as compared to SESS. In agreement with these results, our analysis of cell proliferation showed that the epithelial layer of ESS was able to actively proliferate at all culture times, whereas SESS epithelium proliferation rate tended to decrease with the culture time. Again, these results support the idea that the presence of stromal cells is associated with a more differentiated epithelial layer with lower proliferation rate [25, 26].

Then, we characterized the stromal layer of ESS and SESS. In this regard, we first found that our SESS model efficiently allowed stromal cell integration. Strikingly, stromal cells preferentially accumulated on the stromal compartment in direct contact with the epithelium (Z1 zone), and cells were scattered and less abundant in the rest of the stroma (Z2 zone). Although the typical rete ridges and papillae of the native tissues are not found in tissue substitutes kept ex vivo [27, 28], the fact that the Z1 zone was enriched in stromal cells partially resembles native tissues, in which the papillary dermis typically contains higher amounts of stromal cells and display different morphologies and gene functions [29]. When we performed a histological study of collagen deposition in the artificial stroma of both models, we found that these fibrillar components of the ECM, together with other non-fibrillar components such as VCAN, were mostly absent in all bioengineered tissues, as previously demonstrated in bioengineered tissues kept ex vivo [27]. However, our study revealed the presence of relevant ECM components, especially in the case of proteoglycans. In short, proteoglycans detected with AB were abundant in SESS and very rare in ESS, and their presence was mainly associated with Z1 in SESS, where stromal cells were more abundant. As previously suggested, paracrine factors released by stromal cells may interact with epithelial cells to induce cell and tissue differentiation at both the stromal and epithelial compartments [4, 30]. In addition, it has been demonstrated that stromal cells corresponding to areas in direct contact with the epithelium, corresponding to Z1 in our models, display higher proliferative and synthetic activity than cells isolated from deeper zones of the stroma [31].

One of the most important structures regulating epithelial-stromal interaction in bilayered tissues is the BM, which connects the epithelial and the stromal layers and supports epithelial development [32–34]. The BM consists of a complex network of macromolecules that includes different fibrillar and non-fibrillar molecules, such as collagens, proteoglycans, glycoproteins, and other components, and provides structural support and signaling clues to cells in tissues [2, 12, 35, 36]. Here, we found that epithelial cells were able to synthesize some relevant BM molecules in ESS, such as FN1 and HSPG2, although the PAS staining signal was significantly lower than CTR native tissues. These results are in line with published literature demonstrating that epithelial cells alone can generate specific BM molecules like FN1 [37, 38]. However, the low expression of other relevant components of the BM in ESS and the low PAS signal are consistent with previous works demonstrating that bioengineered human tissues kept in culture are typically devoid of a well-developed BM [9].

In contrast, our results showed that the presence of stromal cells in SESS had a positive effect on BM synthesis and differentiation, with the formation of a more mature structure as compared to ESS that became similar to control tissues when stained with PAS. As in the ESS model, SESS showed an active process of FN1 and HSPG2 synthesis. In addition, we also found that SESS were able to generate abundant amounts of other key components of the BM, including NID1, CH6S, AGRN, COL-IV, and COL-VII, although other components such as LUM were significantly lower than CTR tissues at all development times. Interestingly, three of the BM components were found in SESS at higher levels than control tissues, including FN1, NID1, and COL-VII. Although these results are intriguing and require further research, we may hypothesize that the active process of induction in these substitutes could lead to a rapid synthesis of these components, which could probably be furtherly remodeled at longer development times until reaching the levels of the control native tissues. In this context, previous studies have shown that during wound healing, a transient ECM enriched in FN1 and other molecules is produced, which are then incorporated into a dense network of fibrillar components [4, 39, 40], a phenomenon also observed in our substitutes and resembling what is seen in healthy and functional human skin. Additionally, it is well known that HSPG2 is synthetized early and can facilitate assembling of other BM components [1, 34, 41], whereas CH6S and AGRN contribute to COL-IV and COL-VII deposition [1, 42]. Altogether, these results confirm the capability of fibrin-agarose-based tissue substitutes to develop a BM in culture, and the relevant role of the stromal cells to efficiently induce the formation of this structure in SESS, as previously suggested [33]. As previously reported [21, 43], epithelial cell phenotypes and functions can be modified by the presence of the stromal cells in the SESS models, although epithelial cells retain the potential to synthesize some relevant ECM components in the absence of the stromal cells. Few manuscripts address BM maturation and dermal–epidermal junction formation in artificial skin or bilayered tissue substitutes. However, our comprehensive characterization aligns with previously published studies [44], which also demonstrated the deposition of newly synthesized extracellular matrix components like collagen in an ultrastructurally organized architecture in in vitro tissue-engineered models [44].

Finally, our protein metabolic pathway analysis demonstrated that our human bilayered tissue substitutes allowed the examination of differential mechanistic insights during artificial tissue maturation. Our protein analysis results confirmed the histological results and revealed that important signaling pathways during matrix deposition, such as the TGF-β pathway, were upregulated as development time increases. TGF-β pathway exerts its effects on cell proliferation, differentiation, and modulation of extracellular matrix components [45–47]. Although the interaction between TGF-β and the ECM is complex and it is not completely understood, the activation of the TGF-β pathway is closely related to the deposition of both fibrillar and non-fibrillar components [43, 45]. Various signaling mechanisms activate the TGF-β family, including SMAD-dependent and SMAD-independent routes. Surprisingly, our study found differential TGF-β pathway activation between the two human bilayered tissue substitutes. In particular, SESS showed a high activation of c-Jun protein whereas ESS showed a high activation of SMAD4 protein, both differential mediators of TGF-β pathway. These results are in agreement with the histological findings, as activation of this pathway ultimately leads to the synthesis of ECM components such as collagen and FN1. In this sense, FN1 immunodetection exhibited higher expression in SESS models, which could be potentially linked to TGF-β activation mediated by c-Jun, as demonstrated by Hocevar et al. in previous studies [45]. These findings also align with previous research indicating that TGF-β can induce FN1 synthesis independently of SMAD4, requiring the activation of c-Jun [45–47]. Moreover, we also detected dual activation of TGF-β proteins in SESS by SMAD proteins, highlighting the complexity and synergistic effects of various components of the TGF-β pathway when both stromal and epithelial cells are present.

These results support the feasibility of studying the mechanistic insights of complex biological processes using our human bilayered tissue substitutes. They underscore the critical role of fibroblasts in orchestrating cellular behavior and matrix synthesis, emphasizing the complex interplay between epithelial and stromal components in tissue-engineered substitutes. This mechanistic insight highlights the potential of our model as a valuable tool for further exploration of tissue development and regenerative processes.

## Conclusions

In summary, our study reveals the potential usefulness of the bioengineered bilayered human tissue substitutes to reproduce the process of ECM maturation that takes place in human tissues and supports the use of these tissue substitutes to carry out ex vivo analyses of tissue development without the need of using native tissues. Our results allow us to state that epithelial-stromal interaction is crucial for ECM development and differentiation, especially regarding BM components. Through a time-course analysis, we revealed the sequential synthesis of several ECM molecules, highlighted the differences arising from the inclusion of fibroblasts, and contributed to understand the intricate cell behaviors governing the ECM maturation process. While this study opens new avenues for understanding ECM dynamics, continued research is required to unravel the full spectrum of interactions and mechanisms that drive ECM maturation in tissue-engineered bilayered substitutes.

## Methods

### Cell sources

Generation of both ESS and SESS required the use of different cell types. First, we generated primary cell cultures of dermal fibroblasts from human foreskin specimens as previously reported [11, 12]. In brief, tissues were washed in Dulbecco’s Phosphate Buffered Saline (PBS, Sigma-Aldrich, D8662) containing antibiotics and immediately transferred at 4 °C to the laboratory. Tissues were then trimmed in small fragments and digested overnight at 37 °C in a collagenase cocktail (2 mg/ml of *Clostridium histolyticum* collagenase I, Gibco BRL Life Technologies, Karlsruhe, Germany) in Dulbecco’s Modified Eagle’s Medium (DMEM) (Merck, Darmstadt, Germany). Detached fibroblasts were harvested by centrifugation and cultured in DMEM supplemented with 10% fetal bovine serum (FBS) and 1% of a commercial antibiotic–antimycotic solution (all these reagents, from Merck). As a source of epithelial cells, we used a commercial cell culture of normal human keratinocytes (CRL-4048) (American Type Culture Collection -ATCC-, Manassas, VA). Epithelial cells were cultured using QC medium, consisting of a 3:1 mixture of DMEM and Ham-F12 Nutrient Mixture supplemented with 10% FBS, 1% antibiotic–antimycotic solution, 24 μg/ml adenine, 5 μg/ml insulin, 10 ng/ml epidermal growth factor, 1.3 ng/ml triiodothyronine, and 0.4 μg/ml hydrocortisone (all of them from Merck). Cells were cultured using standard culture conditions at 37 °C with 5% CO_2_ in a cell incubator, and media were renewed every 3 days.

### Generation of human tissue substitutes

In the present work, we generated ESS and SESS using fibrin-agarose biomaterials following previously described protocols [12, 48]. Briefly, a stroma substitute was generated by combining a source of fibrin, obtained from human plasma donors and melted type VII agarose diluted in PBS. In brief, to generate a stroma substitute of 0.5 ml of volume, 380 μL of human plasma were mixed with 62.5 μL of supplemented DMEM, 7.5 μL of tranexamic acid (Amchafibrin, Fides Ecopharma, Spain), 25 μL of CaCl_2_ 2%, and 25μL of agarose VII 2%. In the case of the SESS, the 62.5 μL of supplemented DMEM contained human stromal cells to obtain a final cell density in the stromal substitute of 660,000 cells/ml, whereas acellular suplemented DMEM was used to generate the ESS. In both cases, the mixture was carefully mixed and poured in 12-well porous inserts until its complete jellification at 37 °C. Then, 55,000 keratinocytes were seeded on top of each artificial stroma, and tissue substitutes were kept submerged in QC medium for 48 h. Then, the air–liquid culture technique was used for 12 additional days to induce maturation and stratification of the epithelial layer. Tissues were cultured using standard culture conditions at 37 °C and 5% CO_2_ in a cell incubator. Samples were taken for analysis after 2, 7, and 14 days of follow-up.

### Histological, histochemical, and immunohistochemical analyses

To study the sequential maturation process that takes place on the ECM of the tissue substitutes, a comprehensive histochemical and immunohistochemical analysis was performed. Tissues were fixed for 24 h in 4% formaldehyde, dehydrated and embedded in paraffin using routine protocols. Then, 5-μm sections were obtained for histological analysis.

To evaluate cell distribution and general morphology of the epithelial and stromal layers, HE staining analyses were first performed using routine laboratory staining techniques. Then, in order to evaluate the presence and distribution of relevant fibrillar and non-fibrillar ECM components, histochemical analyses were carried out. For the fibrillar components, the histochemical method of PS (for collagen fibers) was used as previously reported [49]. In brief, after sample deparaffinization and rehydration slides were incubated in sirius red F3B for 30 min and counterstaining with Harris’s hematoxylin for 5 min. Analysis of non-fibrillar components, including glycoproteins and proteoglycans, was carried out by PAS and AB histochemistry, as described elsewhere [35]. In brief, samples were rehydrated and for PAS they were incubated in 0.5% periodic acid solution for 5 min, followed by incubation in Schiff reagent for 15 min and counterstaining with Harris hematoxylin for 1 min, whereas for AB slides were incubated in alcian blue working solution for 30 min and counterstained with nuclear fast red for 1 min.

Then, we characterized cell phenotypes and cell proliferation by using indirect immunohistochemical techniques for cytokeratins: PCK, KRT5, and KRT10; cell adhesions molecules (CLDN); stromal markers (VIM); and proliferation markers (PCNA and MKI67). Immunohistochemistry was also used to identify relevant components of the ECM and BM, including several types of collagens (COL-I, COL-IV and COL-VII), HSPG2, AGRN, VCAN, CH6S, LUM, NID1, and FN1. In brief, slides were dewaxed and rehydrated, and antigen retrieval was performed as specified in Table 1. Then, endogenous peroxidase was quenched with H_2_O_2_ (Panreac Química S.L.U.), and unspecific binding sites were blocked with a solution containing casein and horse serum (Vector Laboratories, Burlingame, CA, USA). Samples were incubated overnight with primary antibody, and before ready-to-use secondary antibodies incubation, samples were washed carefully. Secondary antibody was labeled with peroxidase (Vector Laboratories) and a diaminobenzidine (DAB) substrate kit (Vector Laboratories). Finally, samples were briefly counterstained with Harris hematoxylin (Thermo Fisher Scientific, Waltham, MA) for 15 s, followed by 3 min in tap water, and coverslipped. The specific conditions, antibody references, and technical specifications are shown in Table 1, and normal, native human skin samples were used as a CTR for the histochemical and immunohistochemical analyses.

**Table 1:** Primary antibodies and conditions used for the immunohistochemical analyses described in this work

Antibody	Dilution	Antigen retrieval method	Reference
AGRN	1:500	Citrate buffer (pH 6)	Abbexa, Abx 037897
CASP7	1:100	Citrate buffer (pH 6)	Abcam, Ab 69540
CLDN	Prediluted	Citrate buffer (pH6)	Master Diagnostica, MAD-000523QD
COL-I	1:250	Citrate buffer (pH 6)	Abcam, Ab 34710
COL-IV	Prediluted	EDTA buffer (pH8) + pepsin 10 min 37 °C	Master Diagnostica, MAD-000733QD
COL-VII	1:50	EDTA buffer (pH8)	Novus Biologicals, NBP2-37,900
CH6S	1:250	Chondroitinase 60 min 37 °C	Sigma-Aldrich, MAB2035
FN1	1:250	EDTA buffer (pH8)	Invitrogen, 11981
HSPG2	1:200	EDTA buffer (pH8)	Abbexa, Abx 103,270
KRT5	Prediluted	Citrate buffer (pH6)	Master Diagnostica, MAD-000481QD
KRT10	Prediluted	Citrate buffer (pH6)	Master Diagnostica, MAD-000535QD
LUM	1:100	Citrate buffer (pH6)	R&D Systems, AF 2846
MKI67	Prediluted	EDTA buffer (pH8)	Master Diagnostica, MAD-000310QD
NID1	1:30	EDTA buffer (pH8)	R&D Systems, AF2570
PCK	Prediluted	EDTA buffer (pH8)	Master Diagnostica, MAD-001000QD
PCNA	Prediluted	EDTA buffer (pH8)	Master Diagnostica, MAD-000903QD
VCAN	1:100	Chondroitinase 60 min 37 °C	Abcam, Ab 19345
VIM	1:200	Citrate buffer (pH6)	Sigma-Aldrich, V6389

### Protein metabolic pathway analysis

The Human Phosphorylation Multi-Pathway Profiling Array C55 (RayBiotech, AAH-PPP-1–4, Atlanta, United States) was used to measure the expression level of phosphorylated proteins typically found in the TGF-β metabolic pathway, involved in cell proliferation, tissue homeostasis, and ECM deposition. This array kit is a membrane-based sandwich immunoassay able to quantify 7 phosphorylated human proteins simultaneously: ATF2, ATM, C-Fos, c-Jun, SMAD1, SMAD2, SMAD4, SMAD5.

First, protein extraction was performed following standardized protocols supplied by manufacturer. Briefly, a total of 5 samples of each model and time condition were subjected to protein extraction protocol. GenElute RNA/DNA/Protein Plus Purification Kit (Sigma-Aldrich, RDP300-50RXN, St Louis, Missouri, United States) was used to obtain the protein content used in the multi-pathway profiling array. For this, samples were mechanical digested under a liquid nitrogen bath and lysis buffer. Then, samples were collected, and DNA, RNA, and proteins were purified through different affinity columns. Finally, protein content was quantified using Protein Quantification Kit-Rapid (Sigma-Aldrich, 51,254−1KT, St Louis, Missouri, United States), and a concentration of 500 μg/ml was used to perform the multi-pathway profiling array.

Then, protein detection was also performed following manufacturers recommendations. The antibody-loaded nitrocellulose membranes were incubated with the different protein samples overnight. After washing steps, membranes were incubated with a detection antibody cocktail. Finally, nitrocellulose membranes were incubated with HRP conjugate and chemiluminescence detection reagents were added and incubated for 2 min at room temperature prior to X-ray film exposure.

### Quantification and statistical analyses

Quantitative analyses were performed using the ImageJ software (version 1.53 k, National Institute of Health, Bethesda, MD, USA) as detailed in previous studies [48, 50–52]. First, in SESS stroma, we identified two different cellular enriched zones, Z1, immediately below the epithelium zone, and Z2, corresponding to the majority of the artificial stroma. Then, cell density was calculated by counting cell nuclei in a controlled, standard area of 0.01mm^2^ (for the epithelial compartment) or 0.02 mm^2^ and 0.05 mm^2^ (for the Z1 and Z2 zones of the stromal compartment, respectively). The cell proliferation rate was determined by quantifying the percentage of cells showing positive immunostaining signal for PCNA and MKI67 in each of these regions.

Then, to evaluate the staining intensity for the histochemical and immunohistochemical analyses of ECM components, we first selected the appropriate color signal using the threshold function of ImageJ. Once the positive signal was isolated, the staining signal intensity was quantified by using the automatic measurement tool of the software. For the epithelial and stromal components, quantification was performed in a square area of 84 × 84 pixels, whereas BM components were quantified using the point tool of the software. Ten measurements were obtained per study group. Finally, all values were normalized to the normal skin samples used as controls, whose signal intensity was considered as 100% expression values.

Finally, to quantify protein metabolic pathways activation, X-ray films were scanned, and signal intensity was quantified at each spot with the ImageJ software in two technical replicates included in each membrane. The results were normalized to positive and negative controls included in the array.

Averages and standard deviations were calculated for each sample and each analysis variable. Comparisons were then performed using Mann-Whitey statistical tests, as most distributions demonstrated not to accomplish parametrical standards. Correlation analyses were performed using Kendall tau statistical tests. All statistical analyses were carried out using the Real Statistics Resource Pack software (Release 7.2) (Dr. Charles Zaiontz, Purdue University, West Lafayette, IN, USA) available at (https://www.real-statistics.com/). Statistical *p* values were corrected for all comparisons and considered statistically significant below *0.001*.

## Supplementary Information

Additional File 1: Table S1. Zone dependent statistical *p* values results of the comparisons of Zone 1 (Z1) versus Zone 2 (Z2) for each time point (2, 7 and 14 days) and each group (the full-thickness stromal-epithelial substitutes (SESS) and the epithelial substitutes (ESS)) for each variable using the Mann–Whitney test. Statistically significant *p* values below 0.001 are labeled in bold and considered statistically significant.

Additional File 2: Table S2. Time and group dependent statistical *p* values of the comparisons of different time points and of the full-thickness stromal-epithelial substitutes (SESS) versus epithelial substitutes (ESS) for each variable using the Mann–Whitney test and the Kendall correlation test. Statistically significant *p* values below 0.001 are labeled in bold and considered statistically significant.

## Data Availability

The data that support the findings of this study are openly available at ZENODO repository 10.5281/zenodo.10432738 [53]. Data are available under the terms of the CC-BY 4.0 Creative Commons Attribution-NonCommercial 4.0 International license (CC BY-NC 4.0).

## Abbreviations

AB: Alcian blue
AGRN: Agrin
BM: Basement membrane
CASP7: Caspase 7
CH6S: Chondroitin-6-sulfate
CLDN: Claudin
COL-I: Collagen type I
COL-IV: Collagen type IV
COL-VII: Collagen type VII
CTR: Control group
DAB: Diaminobenzidine
DMEM: Dulbecco’s Modified Eagle’s Medium
ECM: Extracellular matrix
ESS: Epithelial substitutes
FBS: Fetal bovine serum
FN1: Fibronectin 1
HE: Hematoxylin-eosin
HSPG2: Perlecan
KRT5: Cytokeratin 5
KRT10: Cytokeratin 10
LUM: Lumican
NID1: Nidogen 1
PAS: Periodic acid-Schiff
PBS: Dulbecco’s Phosphate Buffered Saline
PCK: Pancytokeratin
PS: Picrosirius red
SESS: Full-thickness stromal-epithelial substitutes
TGF-β: Transforming growth factor-β
VCAN: Versican
VIM: Vimentin
Z1: Zone 1
Z2: Zone 2

## References

[1] Rousselle P, Montmasson M, Garnier C. Extracellular matrix contribution to skin wound re-epithelialization. Matrix Biol J Int Soc Matrix Biol. 2019;75–76:12–26. 10.1016/j.matbio.2018.01.002 29330022

[2] Theocharis AD, Skandalis SS, Gialeli C, Karamanos NK. Extracellular matrix structure. Adv Drug Deliv Rev. 2016;97:4–27. 26562801 10.1016/j.addr.2015.11.001

[3] García-García ÓD, Chato-Astrain J, Hochuli AHD, Pozzobon M, Carriel V. Decellularized tissue-derived materials for grafts development. In: Maia FRA, Oliveira JM, Reis RL, editors. Handbook of the Extracellular Matrix: Biologically-Derived Materials. Cham: Springer International Publishing; 2023. p. 1–35.

[4] Diller RB, Tabor AJ. The role of the extracellular matrix (ECM) in wound healing: a review. Biomimetics. 2022;7:87. 35892357 10.3390/biomimetics7030087 PMC9326521

[5] Smith MM, Melrose J. Proteoglycans in normal and healing skin. Adv Wound Care. 2015;4:152–73. 10.1089/wound.2013.0464 PMC4352701 25785238

[6] Hu M, Ling Z, Ren X. Extracellular matrix dynamics: tracking in biological systems and their implications. J Biol Eng. 2022;16:13. 35637526 10.1186/s13036-022-00292-x PMC9153193

[7] Waschke J, Koch M, Kürten S, Schulze-Tanzil G, Spittau B. Sobotta. Texto de anatomía. Elsevier Health Sciences; 2018.

[8] Liu J, Mao JJ, Chen L. Epithelial-mesenchymal interactions as a working concept for oral mucosa regeneration. Tissue Eng Part B Rev. 2011;17:25–31. 21062224 10.1089/ten.teb.2010.0489 PMC3045573

[9] Martin-Piedra MA, Carmona G, Campos F, Carriel V, Fernández-González A, Campos A, et al. Histological assessment of nanostructured fibrin-agarose skin substitutes grafted in burnt patients. A time-course study. Bioeng Transl Med. 2023;8:e10572. 38023713 10.1002/btm2.10572 PMC10658487

[10] Egea-Guerrero JJ, Carmona G, Correa E, Mata R, Arias-Santiago S, Alaminos M, et al. Transplant of tissue-engineered artificial autologous human skin in Andalusia: an example of coordination and institutional collaboration. Transplant Proc. 2019;51:3047–50. 31627920 10.1016/j.transproceed.2019.08.014

[11] Ruiz-López J, Cardona JC, Garzón I, Pérez MM, Alaminos M, Chato-Astrain J, et al. Optical Behavior of human skin substitutes: absorbance in the 200–400 nm UV range. Biomedicines. 2022;10:1640. 35884945 10.3390/biomedicines10071640 PMC9313464

[12] Carriel V, Garzón I, Jiménez J-M, Oliveira A-C-X, Arias-Santiago S, Campos A, et al. Epithelial and stromal developmental patterns in a novel substitute of the human skin generated with fibrin-agarose biomaterials. Cells Tissues Organs. 2012;196:1–12. 22146480 10.1159/000330682

[13] Ionescu AM, Chato-Astrain J, Cardona Pérez JC, Campos F, Pérez Gómez M, Alaminos M, et al. Evaluation of the optical and biomechanical properties of bioengineered human skin generated with fibrin-agarose biomaterials. J Biomed Opt. 2020;25:1–16. 32383372 10.1117/1.JBO.25.5.055002 PMC7203517

[14] Cardona J de la C, Ionescu A-M, Gómez-Sotomayor R, González-Andrades M, Campos A, Alaminos M, et al. Transparency in a fibrin and fibrin-agarose corneal stroma substitute generated by tissue engineering. Cornea. 2011;30:1428–35. 21934491 10.1097/ICO.0b013e31821bdfd4

[15] Viñuela-Prieto JM, Sánchez-Quevedo MC, Alfonso-Rodríguez CA, Oliveira AC, Scionti G, Martín-Piedra MA, et al. Sequential keratinocytic differentiation and maturation in a three-dimensional model of human artificial oral mucosa. J Periodontal Res. 2015;50:658–65. 25470318 10.1111/jre.12247

[16] Jaimes-Parra BD, Valle-Díaz de la Guardia F, Arrabal-Polo MÁ, Herrera-Imbroda B, Lara MF, Machuca-Santa-Cruz F-J, et al. Ex vivo construction of a novel model of bioengineered bladder mucosa: a preliminary study. Int J Urol Off J Jpn Urol Assoc. 2016;23:85–92. 10.1111/iju.12963 26502190

[17] Jain P, Rauer SB, Möller M, Singh S. Mimicking the natural basement membrane for advanced tissue engineering. Biomacromol. 2022;23:3081–103. 10.1021/acs.biomac.2c00402 PMC9364315 35839343

[18] Blanco-Elices C, Morales-Álvarez C, Chato-Astrain J, González-Gallardo C, Ávila-Fernández P, Campos F, et al. Development of stromal differentiation patterns in heterotypical models of artificial corneas generated by tissue engineering. Front Bioeng Biotechnol. 2023;11:1124995. 37034263 10.3389/fbioe.2023.1124995 PMC10076743

[19] Chato-Astrain J, Chato-Astrain I, Sánchez-Porras D, García-García Ó-D, Bermejo-Casares F, Vairo C, et al. Generation of a novel human dermal substitute functionalized with antibiotic-loaded nanostructured lipid carriers (NLCs) with antimicrobial properties for tissue engineering. J Nanobiotechnology. 2020;18:174. 33228673 10.1186/s12951-020-00732-0 PMC7686763

[20] Bilodeau C, Shojaie S, Goltsis O, Wang J, Luo D, Ackerley C, et al. TP63 basal cells are indispensable during endoderm differentiation into proximal airway cells on acellular lung scaffolds. NPJ Regen Med. 2021;6:12. 33674599 10.1038/s41536-021-00124-4 PMC7935966

[21] Okazaki M, Yoshimura K, Suzuki Y, Harii K. Effects of subepithelial fibroblasts on epithelial differentiation in human skin and oral mucosa: heterotypically recombined organotypic culture model. Plast Reconstr Surg. 2003;112:784–92. 12960859 10.1097/01.PRS.0000069710.48139.4E

[22] Kobayashi K, Nomoto Y, Suzuki T, Tada Y, Miyake M, Hazama A, et al. Effect of fibroblasts on tracheal epithelial regeneration in vitro. Tissue Eng. 2006;12:2619–28. 16995795 10.1089/ten.2006.12.2619

[23] Evtushenko NA, Beilin AK, Kosykh AV, Vorotelyak EA, Gurskaya NG. Keratins as an inflammation trigger point in epidermolysis bullosa simplex. Int J Mol Sci. 2021;22:12446. 34830328 10.3390/ijms222212446 PMC8624175

[24] Kim Y, Lim K-M. Skin barrier dysfunction and filaggrin. Arch Pharm Res. 2021;44:36–48. 33462753 10.1007/s12272-021-01305-x

[25] Ruijtenberg S, van den Heuvel S. Coordinating cell proliferation and differentiation: antagonism between cell cycle regulators and cell type-specific gene expression. Cell Cycle. 2016;15:196–212. 26825227 10.1080/15384101.2015.1120925 PMC4825819

[26] Iida T, Takami Y, Yamaguchi R, Shimazaki S, Harii K. Development of a tissue-engineered human oral mucosa equivalent based on an acellular allogeneic dermal matrix: a preliminary report of clinical application to burn wounds. Scand J Plast Reconstr Surg Hand Surg. 2005;39:138–46. 16019745 10.1080/0284431051006376

[27] Garzon I, Serrato D, Roda O, Del Carmen S-Q, Gonzales-Jaranay M, Moreu G, et al. In vitro cytokeratin expression profiling of human oral mucosa substitutes developed by tissue engineering. Int J Artif Organs. 2009;32:711–9. 19943232 10.1177/039139880903201002

[28] Martin-Piedra MA, Alfonso-Rodriguez CA, Zapater A, Durand-Herrera D, Chato-Astrain J, Campos F, et al. Effective use of mesenchymal stem cells in human skin substitutes generated by tissue engineering. Eur Cell Mater. 2019;37:233–49. 30924522 10.22203/eCM.v037a14

[29] Wu S, Rietveld M, Hogervorst M, de Gruijl F, van der Burg S, Vermeer M, et al. Human papillary and reticular fibroblasts show distinct functions on tumor behavior in 3D-organotypic cultures mimicking melanoma and HNSCC. Int J Mol Sci. 2022;23:11651. 36232952 10.3390/ijms231911651 PMC9570214

[30] Russo B, Brembilla NC, Chizzolini C. Interplay between keratinocytes and fibroblasts: a systematic review providing a new angle for understanding skin fibrotic disorders. Front Immunol. 2020;11:648. 32477322 10.3389/fimmu.2020.00648 PMC7232541

[31] Rippa AL, Kalabusheva EP, Vorotelyak EA. Regeneration of dermis: scarring and cells involved. Cells. 2019;8:607. 31216669 10.3390/cells8060607 PMC6627856

[32] Chan LS. Human skin basement membrane in health and in autoimmune diseases. Front Biosci J Virtual Libr. 1997;2:d343-352. 10.2741/a196 9232815

[33] Lee D-Y, Cho K-H. The effects of epidermal keratinocytes and dermal fibroblasts on the formation of cutaneous basement membrane in three-dimensional culture systems. Arch Dermatol Res. 2005;296:296–302. 15650892 10.1007/s00403-004-0529-5

[34] Yurchenco PD. Integrating activities of laminins that drive basement membrane assembly and function. Curr Top Membr. 2015;76:1–30. 26610910 10.1016/bs.ctm.2015.05.001

[35] Sánchez-Porras D, Varas J, Godoy-Guzmán C, Bermejo-Casares F, San Martín S, Carriel V. Histochemical and immunohistochemical methods for the identification of proteoglycans. Methods Mol Biol Clifton NJ. 2023;2566:85–98. 10.1007/978-1-0716-2675-7_7 36152244

[36] Godoy-Guzmán C, Nuñez C, Orihuela P, Campos A, Carriel V. Distribution of extracellular matrix molecules in human uterine tubes during the menstrual cycle: a histological and immunohistochemical analysis. J Anat. 2018;233:73–85. 29663371 10.1111/joa.12814 PMC5987832

[37] Alitalo K, Kuismanen E, Myllylä R, Kiistala U, Asko-Seljavaara S, Vaheri A. Extracellular matrix proteins of human epidermal keratinocytes and feeder 3T3 cells. J Cell Biol. 1982;94:497–505. 6182145 10.1083/jcb.94.3.497 PMC2112228

[38] Clark RA, Nielsen LD, Howell SE, Folkvord JM. Human keratinocytes that have not terminally differentiated synthesize laminin and fibronectin but deposit only fibronectin in the pericellular matrix. J Cell Biochem. 1985;28:127–41. 2416765 10.1002/jcb.240280206

[39] Kurkinen M, Vaheri A, Roberts PJ, Stenman S. Sequential appearance of fibronectin and collagen in experimental granulation tissue. Lab Investig J Tech Methods Pathol. 1980;43:47–51. 6993786

[40] Lenselink EA. Role of fibronectin in normal wound healing. Int Wound J. 2013;12:313–6. 23742140 10.1111/iwj.12109 PMC7950333

[41] Oldberg A, Ruoslahti E. Interactions between chondroitin sulfate proteoglycan, fibronectin, and collagen. J Biol Chem. 1982;257:4859–63. 6802842

[42] Tracy LE, Minasian RA, Caterson EJ. Extracellular matrix and dermal fibroblast function in the healing wound. Adv Wound Care. 2016;5:119–36. 10.1089/wound.2014.0561 PMC4779293 26989578

[43] Yamaguchi Y, Hearing VJ, Itami S, Yoshikawa K, Katayama I. Mesenchymal-epithelial interactions in the skin: aiming for site-specific tissue regeneration. J Dermatol Sci. 2005;40:1–9. 16157476 10.1016/j.jdermsci.2005.04.006

[44] Auxenfans C, Fradette J, Lequeux C, Germain L, Kinikoglu B, Bechetoille N, et al. Evolution of three dimensional skin equivalent models reconstructed in vitro by tissue engineering. Eur J Dermatol EJD. 2009;19:107–13. 19106039 10.1684/ejd.2008.0573

[45] Hocevar BA, Brown TL, Howe PH. TGF-beta induces fibronectin synthesis through a c-Jun N-terminal kinase-dependent, Smad4-independent pathway. EMBO J. 1999;18:1345–56. 10064600 10.1093/emboj/18.5.1345 PMC1171224

[46] Derynck R, Zhang YE. Smad-dependent and Smad-independent pathways in TGF-beta family signalling. Nature. 2003;425:577–84. 14534577 10.1038/nature02006

[47] Verrecchia F, Mauviel A. Transforming growth factor-beta signaling through the Smad pathway: role in extracellular matrix gene expression and regulation. J Invest Dermatol. 2002;118:211–5. 11841535 10.1046/j.1523-1747.2002.01641.x

[48] Ortiz-Arrabal O, Irastorza-Lorenzo A, Campos F, Martín-Piedra MÁ, Carriel V, Garzón I, et al. Fibrin and marine-derived agaroses for the generation of human bioartificial tissues: an ex vivo and in vivo study. Mar Drugs. 2023;21:187. 36976236 10.3390/md21030187 PMC10058299

[49] Vela-Romera A, Carriel V, Martín-Piedra MA, Aneiros-Fernández J, Campos F, Chato-Astrain J, et al. Characterization of the human ridged and non-ridged skin: a comprehensive histological, histochemical and immunohistochemical analysis. Histochem Cell Biol. 2019;151:57–73. 30099600 10.1007/s00418-018-1701-x PMC6328512

[50] Chato-Astrain J, Roda O, Sánchez-Porras D, Miralles E, Alaminos M, Campos F, et al. Peripheral nerve regeneration through nerve conduits evokes differential expression of growth-associated protein-43 in the spinal cord. Neural Regen Res. 2023;18:1852–6. 36751816 10.4103/1673-5374.363180 PMC10154484

[51] García-García ÓD, El Soury M, González-Quevedo D, Sánchez-Porras D, Chato-Astrain J, Campos F, et al. Histological, biomechanical, and biological properties of genipin-crosslinked decellularized peripheral nerves. Int J Mol Sci. 2021;22:E674. 10.3390/ijms22020674 PMC7826762 33445493

[52] Ortiz-Arrabal O, Carmona R, García-García Ó-D, Chato-Astrain J, Sánchez-Porras D, Domezain A, et al. Generation and evaluation of novel biomaterials based on decellularized sturgeon cartilage for use in tissue engineering. Biomedicines. 2021;9:775. 34356839 10.3390/biomedicines9070775 PMC8301329

[53] Ávila-Fernandez P, Etayo-Escanilla M, Sánchez-Porras D, Fernandez-Valadés R, Campos F, Garzon I, Carriel V, Alaminos M, García-García ÓD, Chato-Astrain J. Quantitative results of the analysis of relevant components of artificial bilayered substitutes developed by tissue engineering. 2023. https://zenodo.org/records/10432738.

