# Supplementary material for: Spatiotemporal characterization of extracellular matrix maturation in human artificial stromal-epithelial tissue substitutes
Source: BMC Biol. 2024 Nov 18;22:263. doi: 10.1186/s12915-024-02065-y (PMC11575135; doi:10.1186/s12915-024-02065-y)
Supplement: Supplementary file 1 — Additional File 1: Table S1. Zone dependent statistical p values results of the comparisons of Zone 1 (Z1) versus Zone 2 (Z2) for each time point (2, 7 and 14 days) and each group (the full-thickness stromal-epithelial substitutes (SESS) and the epithelial substitutes (ESS)) for each variable using the Mann–Whitney test. Statistically significant p values below 0.001 are labeled in bold and considered statistically significant. [file 12915_2024_2065_MOESM1_ESM.docx]

|  |  | **Z1 vs. Z2** | | |
| --- | --- | --- | --- | --- |
|  |  | **2d** | **7d** | **14d** |
| **Cell Density (cells/mm^2^)** | **ESS** | 0.9705 | 0.9705 | 0.9705 |
|  | **SESS** | **p<0.0001** | **p<0.0001** | **0.0001** |
| **PCNA** | **ESS** | 0.9705 | 0.9705 | 0.9705 |
|  | **SESS** | 0.2799 | 0.7959 | 0.1655 |
| **MKI67** | **ESS** | 0.9705 | 0.9705 | 0.9705 |
|  | **SESS** | 0.0185 | **p<0.0001** | 0.0068 |
| **VIM** | **ESS** | 0.9705 | 0.9705 | 0.9705 |
|  | **SESS** | 0.0015 | **0.0003** | 0.3930 |
| **PS** | **ESS** | 0.9705 | 0.9705 | 0.1431 |
|  | **SESS** | 0.2176 | **0.0001** | **p<0.0001** |
| **AB** | **ESS** | 0.4813 | 0.4813 | 0.7394 |
|  | **SESS** | 0.1051 | **p<0.0001** | **p<0.0001** |
| **COL-I** | **ESS** | 0.0147 | 0.9118 | 0.0185 |
|  | **SESS** | 0.3150 | **0.0007** | 0.0115 |
| **VCAN** | **ESS** | 0.1051 | 0.8534 | 0.0288 |
|  | **SESS** | 0.0089 | **p<0.0001** | **p<0.0001** |

**SUPPLEMENTARY TABLE S1:** Zone dependent statistical p values results of the comparisons of Zon1 (Z1) versus Zone 2 (Z2) for each time point (2, 7 and 14 days) and each group (the full-thickness stromal-epithelial substitutes (SESS) and the epithelial substitutes (ESS)) for each variable using the Mann–Whitney test. Statistically significant p values bellow 0.001 are labeled win bold and considered statistically significant.
