# Supplementary material for: Spatiotemporal characterization of extracellular matrix maturation in human artificial stromal-epithelial tissue substitutes
Source: BMC Biol. 2024 Nov 18;22:263. doi: 10.1186/s12915-024-02065-y (PMC11575135; doi:10.1186/s12915-024-02065-y)
Supplement: Supplementary file 2 — Additional File 2: Table S2. Time and group dependent statistical p values of the comparisons of different time points and of the full-thickness stromal-epithelial substitutes (SESS) versus epithelial substitutes (ESS) for each variable using the Mann–Whitney test and the Kendall correlation test. Statistically significant p values below 0.001 are labeled in bold and considered statistically significant. [file 12915_2024_2065_MOESM2_ESM.docx]

|  | | | **ESS** | | | | | | | | | **SESS** | | | | | | | | | | | | | **ESS vs. SESS** | | | |
| --- | --- | --- | --- | --- | --- | --- | --- | --- | --- | --- | --- | --- | --- | --- | --- | --- | --- | --- | --- | --- | --- | --- | --- | --- | --- | --- | --- | --- |
|  | **Marker** | | **2d vs.** | | | **7d vs.** | | **14d vs.** | **Kendall** | **Kendall (pvalue)** | **2d vs.** | | | | **7d vs.** | | | **14d vs.** | | **Kendall** | | **Kendall (pvalue)** | |  | | | |  |
|  |  |  | **7d** | **14d** | **CTR** | **14d** | **CTR** | **CTR** |  |  | **7d** | | **14d** | **CTR** | **14d** | **CTR** | **CTR** | |  | |  | | **2d** | | | **7d** | **14d** |  |
| **EP** | **PCK** | | **p<0.0001** | **p<0.0001** | **p<0.0001** | 0.9705 | **p<0.0001** | **0.0007** | 0.5383 | **0.0002** | **p<0.0001** | | **p<0.0001** | **p<0.0001** | 0.4813 | 0.0011 | 0.0039 | | 0.5057 | | **0.0006** | | **p<0.0001** | | | **0.0001** | 0.0115 |  |
|  | **KRT5** | | **0.0001** | **0.0007** | **p<0.0001** | 0.6305 | **p<0.0001** | **p<0.0001** | 0.5216 | **0.0004** | 0.0011 | | **p<0.0001** | **p<0.0001** | 0.6842 | **p<0.0001** | **p<0.0001** | | -0.5392 | | **0.0002** | | 0.4813 | | | **p<0.0001** | **p<0.0001** |  |
|  | **KRT10** | | 0.0029 | 0.3527 | **p<0.0001** | **p<0.0001** | **p<0.0001** | **p<0.0001** | 0.0167 | 0.9091 | **p<0.0001** | | **p<0.0001** | **p<0.0001** | 0.7959 | **p<0.0001** | **p<0.0001** | | 0.5333 | | **0.0003** | | 0.7959 | | | 0.3527 | 0.0068 |  |
|  | **CLDN** | | **p<0.0001** | **0.0005** | **p<0.0001** | **p<0.0001** | **p<0.0001** | **p<0.0001** | 0.2422 | 0.0979 | 0.6842 | | **p<0.0001** | **p<0.0001** | **0.0007** | **p<0.0001** | **p<0.0001** | | 0.4650 | | 0.0015 | | 0.0068 | | | **0.0001** | 0.0039 |  |
|  | **PCNA** | | 0.1431 | 0.5787 | 0.0011 | 0.1431 | **0.0002** | **0.0005** | 0.0648 | 0.6955 | 0.0068 | | **0.0005** | **0.0005** | 0.0288 | 0.0015 | 0.0524 | | -0.6232 | | **p<0.0001** | | 0.4813 | | | **0.0002** | **p<0.0001** |  |
|  | **MKI67** | | 0.0068 | 0.0015 | 0.3150 | 0.8534 | **0.0007** | **0.0003** | 0.3995 | 0.0062 | **p<0.0001** | | **p<0.0001** | **p<0.0001** | 0.0052 | **0.0001** | 0.0185 | | -0.3572 | | 0.0149 | | **p<0.0001** | | | **p<0.0001** | **p<0.0001** |  |
| **BM** | **PAS** | | 0.0288 | 0.3930 | **p<0.0001** | 0.0185 | **p<0.0001** | **0.0005** | 0.0753 | 0.6074 | **p<0.0001** | | **p<0.0001** | **p<0.0001** | 0.7394 | 0.0052 | 0.0185 | | 0.5044 | | **0.0005** | | 0.1903 | | | **p<0.0001** | **p<0.0001** |  |
|  | **FN1** | | 0.0021 | **p<0.0001** | **0.0007** | **p<0.0001** | 0.0021 | **p<0.0001** | 0.7711 | **p<0.0001** | **p<0.0001** | | **p<0.0001** | 0.0021 | 0.0753 | **0.0003** | **p<0.0001** | | 0.6651 | | **p<0.0001** | | 0.0015 | | | **p<0.0001** | 0.0115 |  |
|  | **NID1** | | 0.1431 | 0.0068 | **p<0.0001** | 0.3527 | **p<0.0001** | **p<0.0001** | -0.3760 | 0.0111 | **p<0.0001** | | **p<0.0001** | **0.0007** | 0.9118 | **0.0002** | **p<0.0001** | | 0.5389 | | **0.0002** | | 0.2176 | | | **p<0.0001** | **p<0.0001** |  |
|  | **CH6S** | | 0.6305 | 0.2176 | **p<0.0001** | 0.2475 | **p<0.0001** | **p<0.0001** | 0.2225 | 0.1280 | **p<0.0001** | | **p<0.0001** | **p<0.0001** | 0.0039 | 0.0232 | 0.2799 | | 0.3524 | | 0.0157 | | 0.4813 | | | **p<0.0001** | **0.0007** |  |
|  | **HSPG2** | | 0.5787 | 0.0232 | 0.0753 | 0.0753 | 0.4359 | 0.2475 | 0.3436 | 0.0184 | 0.0089 | | 0.0524 | 0.5787 | 0.0753 | 0.0232 | 0.0433 | | 0.1972 | | 0.1769 | | 0.5787 | | | 0.0068 | 0.9118 |  |
|  | **AGRN** | | 0.2799 | 0.5288 | **0.0001** | 0.9118 | **0.0001** | **p<0.0001** | -0.1210 | 0.4126 | 0.0115 | | 0.1903 | **0.0001** | 0.2176 | 0.9118 | 0.0524 | | 0.1831 | | 0.2094 | | 0.4359 | | | 0.0021 | 0.0115 |  |
|  | **LUM** | | 0.1051 | 0.6842 | **p<0.0001** | 0.6305 | **p<0.0001** | **p<0.0001** | -0.1939 | 0.1941 | 0.2176 | | 0.2176 | **p<0.0001** | 0.7959 | **p<0.0001** | **p<0.0001** | | 0.2086 | | 0.1537 | | **0.0007** | | | 0.0029 | 0.0029 |  |
|  | **COL-IV** | | 0.0288 | 0.4359 | **p<0.0001** | 0.3527 | **p<0.0001** | **p<0.0001** | 0.1531 | 0.2954 | **p<0.0001** | | **p<0.0001** | **p<0.0001** | 0.6305 | 0.0052 | 0.0011 | | 0.5200 | | **0.0004** | | 0.0433 | | | 0.0115 | 0.0039 |  |
|  | **COL-VII** | | 0.6842 | 0.9705 | **p<0.0001** | 0.3150 | **0.0005** | **p<0.0001** | 0.1040 | 0.4808 | **p<0.0001** | | **p<0.0001** | **p<0.0001** | 0.1051 | 0.0011 | **p<0.0001** | | 0.6742 | | **p<0.0001** | | 0.2475 | | | **p<0.0001** | **p<0.0001** |  |
| **STROMA** | **Cell Density (cells/mm2)** | **Z1** | 0.9705 | 0.9705 | **p<0.0001** | 0.9705 | **p<0.0001** | **p<0.0001** | - | - | **p<0.0001** | | **p<0.0001** | **p<0.0001** | 0.0089 | 0.0011 | 0.0524 | | 0.7316 | | **p<0.0001** | | **p<0.0001** | | | **p<0.0001** | 0.0524 |  |
|  |  | **Z2** | 0.9705 | 0.9705 | **p<0.0001** | 0.9705 | **p<0.0001** | **p<0.0001** | - | - | 0.0052 | | **p<0.0001** | **p<0.0001** | **p<0.0001** | 0.6842 | **p<0.0001** | | 0.7635 | | **p<0.0001** | | **p<0.0001** | | | **p<0.0001** | **p<0.0001** |  |
|  | **PCNA** | **Z1** | 0.9705 | 0.9705 | **0.0002** | 0.9705 | **p<0.0001** | **0.0002** | - | - | 0.6305 | | 0.1903 | **p<0.0001** | 0.0232 | **p<0.0001** | **p<0.0001** | | -0.2738 | | 0.0845 | | **p<0.0001** | | | **p<0.0001** | **p<0.0001** |  |
|  |  | **Z2** | 0.9705 | 0.9705 | **p<0.0001** | 0.9705 | **p<0.0001** | **p<0.0001** | - | - | 0.7394 | | **0.0002** | **p<0.0001** | 0.0052 | **p<0.0001** | **p<0.0001** | | -0.6320 | | **0.0001** | | **p<0.0001** | | | **p<0.0001** | **p<0.0001** |  |
|  | **MKI67** | **Z1** | 0.9705 | 0.9705 | 0.9705 | 0.9705 | **p<0.0001** | 0.9705 | - | - | **p<0.0001** | | **p<0.0001** | **p<0.0001** | 0.5288 | **p<0.0001** | **p<0.0001** | | -0.6077 | | **p<0.0001** | | **p<0.0001** | | | **p<0.0001** | **p<0.0001** |  |
|  |  | **Z2** | 0.9705 | 0.9705 | 0.9705 | 0.9705 | 0.9705 | 0.9705 | - | - | **p<0.0001** | | **p<0.0001** | **p<0.0001** | **p<0.0001** | 0.9705 | **p<0.0001** | | -0.2931 | | 0.0526 | | **p<0.0001** | | | 0.9705 | **p<0.0001** |  |
|  | **VIM** | **Z1** | 0.9705 | 0.9705 | **p<0.0001** | 0.9705 | **p<0.0001** | **p<0.0001** | - | - | 0.0288 | | **p<0.0001** | **0.0005** | **p<0.0001** | 0.0052 | **p<0.0001** | | 0.7150 | | **p<0.0001** | | **p<0.0001** | | | **p<0.0001** | **p<0.0001** |  |
|  |  | **Z2** | 0.9705 | 0.9705 | **p<0.0001** | 0.9705 | **p<0.0001** | **p<0.0001** | - | - | 0.7959 | | 0.0355 | 0.5288 | 0.0433 | 0.1655 | 0.0089 | | 0.3270 | | 0.0249 | | **p<0.0001** | | | **p<0.0001** | **p<0.0001** |  |
|  | **PS** | **Z1** | 0.9705 | 0.1431 | **p<0.0001** | 0.1431 | 0.2799 | **p<0.0001** | - | - | **p<0.0001** | | **p<0.0001** | **p<0.0001** | **0.0005** | **p<0.0001** | **p<0.0001** | | 0.7984 | | **p<0.0001** | | **p<0.0001** | | | **p<0.0001** | **p<0.0001** |  |
|  |  | **Z2** | 0.9705 | 0.9705 | **p<0.0001** | 0.9705 | **p<0.0001** | **p<0.0001** | - | - | 0.7959 | | 0.0115 | **p<0.0001** | 0.0630 | **p<0.0001** | **p<0.0001** | | 0.3268 | | 0.0346 | | 0.0232 | | | 0.1431 | **0.0002** |  |
|  | **AB** | **Z1** | 0.9705 | 0.9705 | **p<0.0001** | 0.9705 | **0.0002** | **p<0.0001** | - | - | **p<0.0001** | | **p<0.0001** | **p<0.0001** | **p<0.0001** | 0.0015 | **p<0.0001** | | 0.8476 | | **p<0.0001** | | **p<0.0001** | | | **p<0.0001** | **p<0.0001** |  |
|  |  | **Z2** | 0.9705 | 0.7394 | **p<0.0001** | 0.7394 | **p<0.0001** | **p<0.0001** | - | - | 0.3930 | | 0.9705 | **p<0.0001** | 0.3150 | **0.0005** | **p<0.0001** | | 0.0173 | | 0.9086 | | **0.0003** | | | 0.0524 | **p<0.0001** |  |
|  | **COL-I** | **Z1** | 0.9705 | 0.7394 | **p<0.0001** | 0.7394 | **0.0005** | **p<0.0001** | 0.0910 | 0.6032 | **0.0003** | | **p<0.0001** | **p<0.0001** | **p<0.0001** | **p<0.0001** | **p<0.0001** | | 0.8158 | | **p<0.0001** | | **p<0.0001** | | | **p<0.0001** | **p<0.0001** |  |
|  |  | **Z2** | 0.1903 | 0.1903 | **p<0.0001** | 0.1230 | **p<0.0001** | **p<0.0001** | 0.1320 | 0.4148 | **0.0007** | | 0.0011 | **p<0.0001** | 0.6842 | **p<0.0001** | **p<0.0001** | | 0.5069 | | **0.0007** | | 0.7959 | | | **p<0.0001** | **0.0001** |  |
|  | **VCAN** | **Z1** | 0.3930 | 0.0147 | **p<0.0001** | 0.0039 | **p<0.0001** | **p<0.0001** | 0.4852 | 0.0018 | 0.0115 | | 0.0068 | **p<0.0001** | 0.6842 | **p<0.0001** | **p<0.0001** | | 0.4136 | | 0.0051 | | **p<0.0001** | | | **p<0.0001** | **p<0.0001** |  |
|  |  | **Z2** | 0.1230 | 0.9705 | **p<0.0001** | 0.2799 | **p<0.0001** | **p<0.0001** | -0.0379 | 0.8117 | 0.0630 | | 0.0185 | **p<0.0001** | 0.5787 | **p<0.0001** | **p<0.0001** | | 0.3593 | | 0.0161 | | **0.0001** | | | **p<0.0001** | **p<0.0001** |  |

**SUPPLEMENTARY TABLE S2:** Time and group dependent statistical p values of the comparisons of different time points and of the full-thickness stromal-epithelial substitutes (SESS) versus epithelial substitutes (ESS) for each variable using the Mann–Whitney test and the Kendall correlation test. Statistically significant p values bellow 0.001 are labeled in bold and considered statistically significant
